# Perceived Effectiveness, Restrictiveness, and Compliance with Containment Measures against the Covid-19 Pandemic: An International Comparative Study in 11 Countries

**DOI:** 10.3390/ijerph18073806

**Published:** 2021-04-06

**Authors:** Irina Georgieva, Tella Lantta, Jakub Lickiewicz, Jaroslav Pekara, Sofia Wikman, Marina Loseviča, Bevinahalli Nanjegowda Raveesh, Adriana Mihai, Peter Lepping

**Affiliations:** 1 Department of Cognitive Science and Psychology, New Bulgarian University, 1618 Sofia, Bulgaria; 2 Department of Nursing Science, University of Turku, 20500 Turku, Finland; tella.lantta@utu.fi; 3 Faculty of Health Sciences, Jagiellonian University Medical College Krakow, 31-008 Kraków, Poland; jakub.lickiewicz@uj.edu.pl; 4 Paramedic Department, Medical College in Prague, 121 08 Prague, Czech Republic; pekarjar@gmail.com; 5 Department of Criminology, University of Gavle, 80176 Gävle, Sweden; Sofia.Wikman@hig.se; 6 Faculty of Medicine, Medical College, Latvian University in Riga, LV-1079 Riga, Latvia; marina.losevica@lu.lv; 7 Department of Psychiatry, Government Medical College, Mysore 570001, India; raveesh6@yahoo.com; 8 Clinical Department of Medicine GE Palade University of Medicine, Pharmacy Science and Technology, 540142 Târgu Mureș, Romania; dradrianamihai@yahoo.com; 9 Centre for Mental Health and Society, Bangor University, Bangor LL57 2DG, UK; peter.lepping@wales.nhs.uk; 10 Mysore Medical College and Research Institute, Karnataka 570001, India

**Keywords:** pandemic, coronavirus, containment measures, effectiveness, restrictiveness, compliance, Covid-19, public health measures, human rights, proportionality principle

## Abstract

National governments took action to delay the transmission of the coronavirus (SARS-CoV-2) by implementing different containment measures. We developed an online survey that included 44 different containment measures. We aimed to assess how effective citizens perceive these measures, which measures are perceived as violation of citizens’ personal freedoms, which opinions and demographic factors have an effect on compliance with the measures, and what governments can do to most effectively improve citizens’ compliance. The survey was disseminated in 11 countries: UK, Belgium, Netherlands, Bulgaria, Czech Republic, Finland, India, Latvia, Poland, Romania, and Sweden. We acquired 9543 unique responses. Our findings show significant differences across countries in perceived effectiveness, restrictiveness, and compliance. Governments that suffer low levels of trust should put more effort into persuading citizens, especially men, in the effectiveness of the proposed measures. They should provide financial compensation to citizens who have lost their job or income due to the containment measures to improve measure compliance. Policymakers should implement the least restrictive and most effective public health measures first during pandemic emergencies instead of implementing a combination of many restrictive measures, which has the opposite effect on citizens’ adherence and undermines human rights.

## 1. Introduction

Pandemics have been occurring at regular intervals throughout human history. On 11 March 2020, the World Health Organization (WHO) declared the rapidly spreading coronavirus outbreak a pandemic. Since then, the virus has spread to nearly every country on the globe. National governments had to take immediate action to delay the transmission of the virus by implementing different containment measures [1], choosing from relatively innocuous measures, such as disease surveillance and hygienic measures, to considerably more restrictive interventions, such as travel restrictions, quarantine, and mandatory self-isolation at home, closures of restaurants, national parks, and schools, or dissolution of Parliaments. Most of the containment measures have an enormous economical and psychological impact on billions of human lives and raise serious ethical and human rights concerns. The temporary shutdown of nonessential businesses has led to unemployment and economic strain [2], containment measures severely impacted economic activity [3], domestic violence causalities have increased during the pandemic [4], and COVID-19 lockdown measures have led to negative mental health outcomes [5]. Social isolation, increased stress, and physical inactivity due to sustained quarantine, stay-at-home orders, closures of parks, gymnasiums, and fitness centers may have compromised the immune system of some people [6]. Suicide rates and mortality rate due to delayed treatment of life-threatening conditions have increased after the lockdown [7,8], and more negative effects are likely to become apparent over time.

Some containment interventions indisputably restrict citizens’ personal freedom and their fundamental human rights to engage in work, education, meet other people, and move freely within the country or to visit other countries, and the right to health or privacy may be affected by use of surveillance technologies [9,10]. In European Union law, the principle of proportionality plays a crucial role in the protection of fundamental rights [11,12], and it is used to assess whether restrictions and measures affecting human rights appropriately respond to legitimate public interests. The concept of proportionality “implies a means–ends relationship between the aims pursued by a specific action of the government and the means employed to achieve this end”. When applying the proportionality principle to national governments’ responses to the Covid-19 pandemic, the negative financial, psychological, and social consequences of these measures should not outweigh the desired outcome, that is, less infected people so as not to burden healthcare facilities and less fatalities. Deciding on which measure to implement without violating the proportionality principle during a rapidly spreading outbreak is a huge challenge, especially when there is insufficient evidence on which measures violate human rights and the effectiveness of each measure [13]. 

A recent study found that closing all educational institutions, limiting gatherings to 10 people or less, and closing face-to-face businesses each reduced transmission considerably, while the additional effect of stay-at-home orders was comparatively small [14]. However, objective estimates of each containment measure’s effectiveness remain a challenging task because governments usually implement a combination of containment measures simultaneously to reduce the virus transmission. To investigate the cause-and-effect relationship in such a complex environment with many confounding factors raises questions about each finding’s validity. Therefore, this study conducted in 11 countries aims to assess citizens’ perception of the effectiveness and restrictiveness of the national containment measures related to the coronavirus epidemic. 

Furthermore, success in diminishing the spread of the disease may largely depend on the compliance of people with the containment measures. Previous studies found that the duration of containment measures plays a crucial role in tackling the spread of the disease as people become less compliant over time [15]. We, therefore, took a further step and explored the relationship of self-reported citizens’ compliance with other factors such as trust in national government and medical care, fear of getting infected, measures’ perceived effectiveness and restrictiveness, experienced stress during the outbreak, time invested in following the news about the coronavirus outbreak, presence of underlying medical conditions, being infected or having family members infected by the virus, opinion about governments’ response to the outbreak, income loss caused by the crisis, provision of financial compensation for the lost income, and different demographic measures. We aim to answer the following questions:

1. How effective do citizens think the different measures are?
2. Which measures are perceived as a violation of citizens’ personal freedom and need a legal balancing?
3. Which opinions and demographic factors have an effect on compliance with measures?
4. What can governments do to improve citizens’ compliance most effectively with measures?

The impact that the COVID-19 epidemic and applied containment measures have on psychological wellbeing of citizens has been widely studied during the year 2020. However, fewer studies have been undertaken to explore citizens’ views of the containment measures. We found numerous research articles reporting some aspects of interest for our study [16,17]. Most studies were small, focused only on a limited number of containment measures (e.g., social distancing and washing hands) and factors associated with adherence to these measures (e.g., coping styles and personal characteristics), or were from a single country. Papers reporting perceived restrictiveness were missing, as were studies exploring the views toward the whole set of containment measures countries have implemented to stop the spread of Covid-19 virus. We found one multi-country study (three countries) exploring citizens’ perceptions about the effectiveness of containment measures against the Covid-19 pandemic and their compliance with these measures [18]. However, this study was descriptive and did not provide information on perceived restrictiveness of the measures. In addition, one survey study, also including citizens from three countries, explored how respondents accepted containment measures and how willing they were to adhere to the measures [19]. However, these were general questions about the measures and did not provide information about specific measures. Based on the evidence, we may conclude that this is the first study providing a multi-country perspective on perceived effectiveness, restrictiveness, and compliance with forty-four containment measures against the Covid-19 pandemic. The importance of monitoring citizens’ compliance, safety, and effectiveness of mitigation measures and to share findings with the international community and WHO is a key recommendation given by the WHO [20]. Our study is one of the largest surveys of Covid-19-related perceptions conducted among citizens at a multi-country level.

## 2. Materials and Methods

### 2.1. Study Design and Participants

An online survey was developed via the online platform Typeform for the use in 11 countries, which was translated by the authors into 9 relevant languages. Initially, the authors stipulated which containment measures to stop the spread of COVID-19 were implemented in the country they represent. Only measures that have been applied in at least two countries were included in the survey, resulting in 44 different containment measures, excluding only measure 3 (Supplementary Materials, Table S1, p. 1). Data were collected via authors’ personal networks and via Facebook advertising. In order to reach more people and to overcome the limited demographic representation on Facebook, we created a website for the project: www.impact-covid19.com (accessed on 15 May 2020). The New Bulgarian University ran paid Facebook marketing campaigns with a budget of 330 EUR (405 USD) per country, targeting all citizens aged 18 and older in the UK, Belgium (Flemish region only), the Netherlands, Bulgaria, the Czech Republic, Finland, India, Latvia, Poland, Romania, and Sweden. The first assessment took place between 26 June and 31 August 2020. Each participant had to agree and give informed consent in order to be able to complete the survey. No identifying details were collected, except that some participants agreed to participate in the follow-up assessment and provided a personal email for contact. They were approached two months later, between 26 August and 18 November 2020. Ethical approvals for the study were obtained in Poland, Bulgaria, Latvia, Romania, and India. 

### 2.2. Procedures

The survey assessed citizens’ demographic characteristics, their way of coping with the pandemic, citizens’ psychological wellbeing, their self-rated compliance with the measures, and their opinion about the effectiveness and restrictiveness of the 44 measures. We used 4 validated instruments to assess post-traumatic stress disorder (PTSD) with Primary Care PTSD Screen for DSM-5 [21], generalized anxiety with Generalized Anxiety Disorder 2-item [22], depression with Patient Health Questionnaire-2 [23], and panic attacks during the pandemic with Panic Disorder Severity Scale—Self-Report [24]. Findings on citizens’ wellbeing will be reported elsewhere. In addition to the validated instruments, the authors reached a consensus on how to formulate relevant questions investigating citizens’ experience with the pandemic. Survey questions reported in this article are described in Table S2 (Supplementary Materials, p. 3).

Not all measures were applied in all countries, so we asked respondents to evaluate the effectiveness of containment measures that have been applied in their country. We asked respondents to assess measures’ restrictiveness and judge their own compliance to containment measures that have affected them personally. 

### 2.3. Statistical Analysis

Data analysis was conducted though SPSS version 23. For this analysis, we used descriptive measures (mean and proportions), analysis of variance (ANOVA), Chi-square test, Spearman nonparametric correlation coefficient (rho), and multivariable regression model with stepwise selection of the variables. The scores of effectiveness, restrictiveness, and compliance measured on an 11-point Likert scale from 0 to 10 and were converted into percentages.

We built a multivariable model for the compliance with each measure. Only two measures that were evaluated during the follow-up assessment (i.e., contact tracing assessment of Covid-19 transmission and mass testing for Covid-19) were excluded from the bivariate and multivariable analyses due to the low number of compliance assessments, respectively n = 175 and n = 108 (Supplementary Materials, Table S4, p. 5). This model’s coefficients represent the effect of one factor when the other factors are present but held constant. Dummy variables for the 10 countries were included in the model to control for country differences. A stepwise procedure was used to select the factors with significant predictive value.

## 3. Results

The first assessment was completed by 9942 people from 11 countries. Some people have completed the survey multiple times, therefore only the first completion was retained, reducing the number of respondents to 9543 unique responses: UK *UK* (N = 653), Belgium *BE* (N = 374), Netherlands (*NL,* N = 864), Bulgaria (*BG,* N = 1868), Czech Republic (*CZ,* N = 723), Finland (*FI,* N = 542), India (*IN,* N = 779), Latvia (*LV,* N = 643), Poland (*PL,* N = 1008), Romania (*RO,* N = 1504), and Sweden (*SE,* N = 585). The follow-up assessment was completed on average 68 days after the first assessment by 1926 respondents. 

Demographic characteristics of respondents are shown in Table S3 (Supplementary Materials, p. 4). Most of the respondents were female (71.4%). The mean age of respondents was 47.5 years, and the majority of them had obtained bachelor, master, or PhD degrees (60%). The majority of the respondents belonged to the group of nonessential staff (76%), followed by other essential staff (12.5%) and medical staff (11.5%). On average, 26% of the sample reported that they have lost their job, or their income was reduced during the pandemic, but only 17.9% of them had received any financial compensation for it. Only 8.5% of the respondents reported to have had Covid-19 symptoms and/or have been tested positive, while 21.8% of them reported to have a family member being infected by the disease. One-third of the respondents (30.5%) had at least one underlying medical condition exposing them to increased risk of severe illness from the Covid-19 infection. 

**Figure 1:**
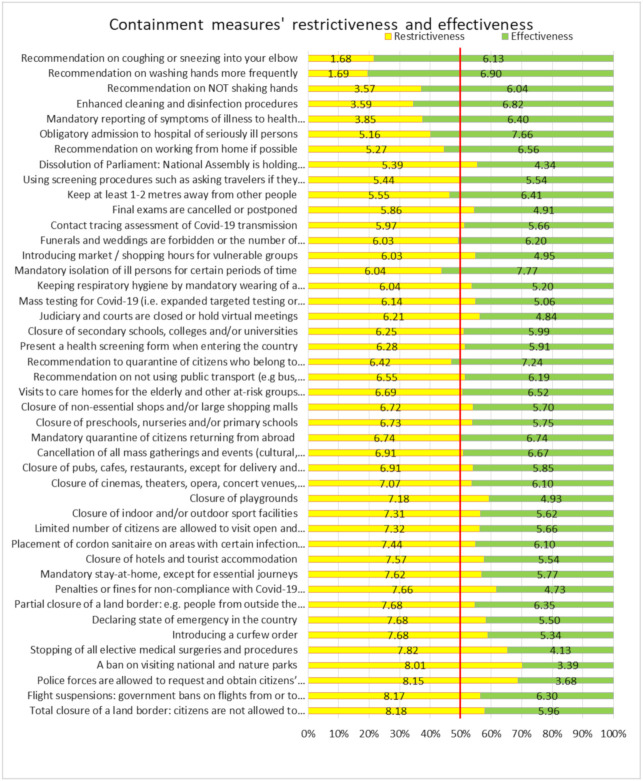
Balance between containment measures’ perceived restrictiveness of personal freedoms and their effectiveness as assessed by study participants.

The majority of respondents (43%) think that the measures taken to prevent the spread of COVID-19 virus have more negative implications than Covid-19 itself, in comparison with 37% of respondents who disagree with this statement (20% neither agreed nor disagreed). The ban on visiting national parks and nature parks was assessed as the most restrictive and least effective measure, calling for legal balancing when implementing this measure (Figure 1). Other measures that require legal balancing, because of violating the human rights to privacy and access to the health services, are respectively “Police forces are allowed to request and obtain citizens’ personal information from internet and telephone providers” and “Stopping of all elective medical surgeries and procedures”. The “Mandatory reporting of symptoms of illness to health authorities” and “Recommendation to quarantine citizens who belong to high-risk groups or who have been in contact with infected people” are also experienced as highly restrictive and involve a significant deprivation of an individual’s liberty in the name of public health. However, their perceived effectiveness greatly outweighed the perceived restrictiveness.

We found that 26% of the respondents have lost their job or have received less income due to the pandemic. Only 18% of them have received any financial COVID-19 compensation, while people who did not receive any compensation had the lowest compliance with the majority (27) of the measures (Supplementary Materials, Table S6, p. 11). This factor had significant impact on only seven containment measures, after controlling for the effect of other factors (Supplementary Materials, Table S7, p. 13).

### 3.1. International Differences

**Figure 2:**
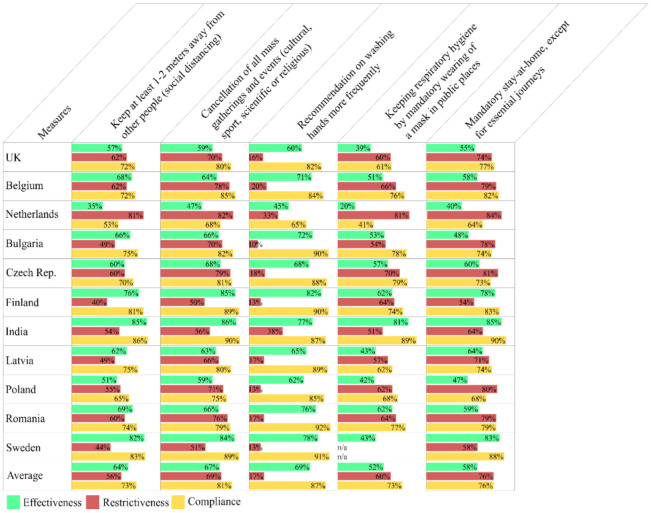
Perceived effectiveness, restrictiveness, and compliance per country for the five measures that have affected the highest number of people personally in all countries.

Variances across countries in perceived effectiveness, restrictiveness, and compliance are shown in Figure 2 and Table S4 (Supplementary Materials, p. 5). Dutch respondents assessed most of the measures as less effective and more restrictive in comparison to respondents from other countries. In contrast to Dutch respondents who also had the lowest compliance with almost all measures, Indian respondents reported the highest compliance. Other differences across countries (Supplementary Materials, Table S3, p. 4) show that Finnish respondents have the most trust that the hospitals in Finland have the resources and expertise to provide the best treatment available to people infected with the coronavirus (7.8), while Polish respondents have the least trust in medical care (2.9). Polish respondents have also the least trust that their Government is taking care of its citizens (1.3), with Finnish people having the most trust in the national Government (6.6). The British, Finnish, and Indian respondents found the national Government’s response to the coronavirus crisis to be the most appropriate (5). Swedish respondents found it the most insufficient (3.9) in contrast to Dutch respondents who assessed the governmental response as the most extreme (7). On the other hand, Dutch respondents found that their Government has been the most factually truthful about the coronavirus outbreak (6.9), while Polish respondents were the most dissatisfied (1.7). Further, Romanian respondents were most stressed by the pandemic (7.7), while Latvian and Czech respondents experienced the lowest stress levels (5.6). Swedish respondents experienced the most fear that they might get infected by the virus (2.5), in comparison with Dutch and Czech respondents being the least fearful (1.6).

### 3.2. Compliance with Containment Measures

Respondents’ compliance with the five measures that have affected the highest number of people personally (78–59%) in all countries is reported in Table 1. The results from the statistical analyses of all 42 measures are reported in Tables S5–S7 (Supplementary Materials, pp. 9–13). 

An example of how to read Table 1 is given below with the first measure reported in the Table “*Keep at least 1–2 m away from other people (social distancing)*”.

This measure affected 78% of study respondents personally. Overall, the average compliance for all countries was 73%, with the Netherlands showing the lowest compliance with 53%, followed by Poland with 65%, and India with the highest compliance at 86%. 

The perceived level of restriction and the perceived effectiveness of social distancing affected the compliance with this measure. People who found this measure more discomforting tended to be less compliant (Spearman’s rho = −0.31), whilst those who found it more effective tended to follow the rule more strictly (rho = 0.61) (Table S5, Supplementary Materials, p. 9).

Other individual factors also influenced how complaint people were with this measure. People who were more compliant watched more news items related to the pandemic; they were older, had a higher education, and trusted hospitals and their government more. They were also more afraid of being infected with the virus and experienced more stress, because of the pandemic. On average, women showed higher compliance rates than men (75% vs. 67%, *p* < *0*.001). People tend to learn from their own experience and from people around them. They tended to be more compliant when somebody in their family got infected (77%), or they had comorbidities themselves, like cardiovascular diseases, diabetes or hepatitis B (77%).

The factor with the biggest effect for keeping to social distancing was fear of getting infected with the virus. When other factors were kept equal, a 10% increase in feeling more fearful brought the compliance up by 10.5% (Table S7, Supplementary Materials, p. 13). Another factor with significant effect was the experienced stress from the outbreak. When people were stressed by the pandemic by an additional 10%, it led to 5% more compliance with this measure. 

The “Reaction of the Government” and the “Trust in the Government” were significantly associated with compliance. When other factors were considered equal, a 10% decrease of an extreme reaction by the Government (moving toward an appropriate reaction) will bring the compliance up by 6%. An increase of 10% in the trust in the Government would bring the compliance up by 4%. 

Perceived effectiveness and restrictiveness of social distancing have a significant effect on the compliance with social distancing rules. A 10% increase in the perceived effectiveness of the measure brings about a 5% increase in compliance. The higher the perceived discomfort from this measure, the lower the compliance, but the effect size is very small: a 10% reduction in discomfort produces a 1% increase in compliance. Further, women followed this rule 3.5% more than men, and older people were 1% more compliant than people a year younger. 

Therefore, in order to increase the rate of compliance with social distancing, the policymakers should focus their efforts on increasing the trust in the Government, avoiding extreme governmental policies, and convincing citizens that social distancing is an effective strategy for preventing the infection. 

## 4. Discussion

Prolonged emergencies lead to a reduction of legal certainty and may cause the rapid and irreversible degradation of the rule of law [9]. With regard to the ongoing COVID-19 pandemic, our findings show which governmental measures are perceived to limit personal freedoms, and thus require constant evaluation and cautious balancing between desired positive outcome and negative consequences. This is particularly important given our finding that, for most measures, perceived high discomfort and restriction of a particular measure influences the compliance with that measure negatively. Perceived restrictiveness, however, has a much smaller effect on compliance than effectiveness, so it seems that people do not mind experiencing a degree of discomfort if they are convinced that the containment measures are effective. This emphasizes the importance of persuading the population of the effectiveness of any measure in any public health campaign. Therefore, the least discomforting and most effective public measures should be implemented first, because a breach of any human right can result not only in economic harms, such us unemployment or loss of insurance or housing, but also in social and psychological harms.

Our findings show more important aspects that should be taken into account when decisions about containment measures are made. More trust in the national government that is perceived to take care of its citizens predicts an increase in compliance with most measures. The same is the case for trust in hospitals to be able to provide good care. We accept that trust in governmental measures and a government’s benign intentions as well as trust in one’s country’s health system do not change overnight and are influenced by many factors. These factors include long-held views, the country’s media, but also the medium- and long-term behaviors of successive governments that allow to build up trust. Whilst trust cannot be developed easily during a pandemic, it is clear that countries where citizens trust their government have an advantage, because they do not need to work quite so hard in order to persuade their citizens of the benefits of the measures they are introducing. Previous studies showed that low levels of political trust are associated with less law compliance within a society [25]. Trusting citizens are also more likely than distrusting citizens to perceive political decisions as being legitimate, even if these decisions are unfavorable to their own particular interests [26], as it is when very restrictive pandemic measures have to be endured. Thus, long-term trustful relations between governments and citizens appear to help in an emergency. This conclusion to maintain and build public trust in public health authorities before, during, and after an influenza pandemic is a key principle of outbreak communication promoted by the WHO [27]. However, the importance of persuading the population of the effectiveness of any measure in any public health campaign has not been set as a key principle in the World Health Organization Outbreak Communication Planning Guide. Based on our findings, we believe that this is fundamental for the successful management and rapid containment of any pandemic influenza. 

Our findings show that Finnish respondents have the most trust in their Government and medical care, which coincides with European data of having the highest trust in the political and legal system in Europe [28]. Maybe this has been the reason for the successful Finnish coronavirus strategy [29], and for having the lowest rate of cumulative cases of Covid-19, 1009.5, and cumulative deaths, 13.3 (per 100,000 population) (2 March 2021 [30]), in comparison with the rest of the European countries involved in our study: Latvia, 4606.8/86.6; Bulgaria, 3565.5/146.9; Romania, 3764.3/95.6; Poland, 4470.2/114.6; UK, 6314.0/185.8; Sweden, 6405.4/125.9; the Netherlands, 6252.5/89.7; Belgium, 6532.2/187.2; Czech Republic, 11,543.7/190.0 [30]. Actually, India has the lowest rate of cumulative cases of Covid-19, 828.5, and cumulative deaths, 11.7 (per 100,000 population), which is not surprising given that Indian and Finnish respondents reported the highest average compliance (80%) with all measures (Table S4, Supplementary Materials, p. 5). This highlights how important it is to follow the public health measures in order to manage pandemic emergencies successfully. To gather feedback from the general public, vulnerable populations, and at-risk groups on attitudes towards the recommended measures and barriers affecting their willingness or ability to comply and to communicate these findings is a key recommendation given by the WHO [20].

In contrast to Finnish respondents, citizens from postcommunist countries like Poland, Bulgaria, and Romania (but not Latvia) had the least trust in public institutions (i.e., government and health system), while their governments had the tendency toward implementing more, highly restrictive containment measures in the beginning of the pandemic (Table S1, Supplementary Materials, p. 1). Interestingly, the UK respondents gave similarly poor ratings for trust in government and government truthfulness, but much better ratings for trust in hospitals. 

Citizens of postcommunist countries have lived in authoritarian regimes, and the restrictions on their personal freedom during the pandemic have brought the old memories and fears from the totalitarian regimes. These countries are still struggling to establish stable political democracies, having the tendency to backslide toward semiauthoritarian and diminished democratic regimes [31,32]. Therefore, it is important that the rule of law and fundamental rights are strictly protected during pandemic emergencies in these countries. As part of political trust, we found that the government’s perceived radicalism of responses plays a significant role in citizens’ compliance. A more balanced or less extreme reaction of the government in dealing with the coronavirus outbreak is associated with an increase in compliance with the majority of the measures. Therefore, it is important that governments carefully consider their decision to implement a combination of many restrictive measures at the same time by weighing up the expected positive results against the negative consequences for the economy and citizens’ mental health, otherwise this may lead to less compliance with the measures. A recent study found that less disruptive and costly containment measures can be as effective as more intrusive and drastic ones (for example, a national lockdown) [33]. 

A number of specific results warrant further discussion. Women follow most containment measures more strictly than men. Without wanting to attempt to explain this finding, it may at least have an influence on government campaign targeting, as it suggests that men need more persuasion than women to follow the measures, which has been concluded earlier [34]. We did not explore respondents’ personal characteristics, but there are findings from Western studies that empathy [35], high agreeableness [36,37], and high emotional stability [37] are associated with compliancy with the measures and with belief that these measures are protecting the citizens’ health, giving some additional explanation to our findings. 

Other important and not unexpected factors are stress from the coronavirus outbreak and an increased fear of getting infected by the virus. The fear of COVID-19 was previously found to predict public health compliance [38]. We also found that fear and stress were both associated with more compliance with most of the containment measures. However, preliminary findings from the same study show that the more stress and fear people experience, the higher the risk of developing a mental illness. Therefore, we do not recommend to policymakers and media to launch public awareness campaigns using fear or threat as a persuasion strategy. This point is emphasized by the finding that an increase in the measure’s perceived effectiveness led to an increase in compliance. This is valid for all measures and clearly points to education about the effectiveness of particular measures as a decisive tool to persuade citizens to be compliant. The fact that this factor was significant with regards to all measures makes it particularly important. 

People with underlying medical conditions that are exposed to a higher risk of severe illness from the coronavirus tend to follow the mandatory cordon sanitaire more strictly and wash their hands more frequently. People who have a family member infected with the virus are also following more strictly some of the measures. They are three times more compliant with not attending indoor and/or outdoor sport facilities and five times more compliant with the rule of mandatory isolation of ill persons for certain periods of time. This points towards an increase in compliance when one’s own health or family members’ lives are directly affected, which is not surprising. 

Interestingly, highly educated people are significantly more compliant with the majority of the measures. Our study did not explore the reasons for these findings, but previous studies found that this is associated with knowledge level about COVID-19 [39,40].

Several limitations need to be noted. First, since the survey was web-based and recruitment was largely through social media, we acknowledge the potential for selection bias. We cannot assume that our study population is representative for the eldest people, who usually do not have access to social media. Second, although the sample size is large and data were collected in eleven countries, Russian-, French-, and Swedish-speaking people were not recruited respectively in Latvia, Belgium, and Finland. Third, the number of completed survey responses was much higher among Bulgarian residents. This is unsurprising since the New Bulgarian University ran the social media marketing campaigns. We also acknowledge that our results might not fully depict citizens’ experience with the measures as most data were collected in August, when countries were easing the lockdown restrictions after the first wave of the COVID-19 pandemic. In addition, we did not take into account in our analysis how severe the pandemic had been in each of the 11 countries. An international study concluded that Italian citizens adhered most strictly with containment measures, because of the severity of the pandemic in their country [18]. However, their study was only descriptive in nature. We acknowledge that the study sample is not representative for the whole of Europe, because it does not include data from any Southern European country. Last but not least, certain public measures (e.g., closure of schools, shops, restaurants, cultural venues, sport facilities, and land borders, and dissolution of Parliaments) compel citizens to follow them rather than giving them a choice. Therefore, citizens’ compliance with these measures might somewhat express citizens’ agreeableness with the measures rather than their adherence. Although study respondents have been explicitly asked to evaluate only the effectiveness, restrictiveness, and compliance of those measures that have been applied in their respective country, we noticed that some respondents have also assessed measures that have not been implemented in their country, suggesting that citizens were not well informed about national policies on the Covid-19 pandemic.

We hope that the findings from this study will be taken into account when each government evaluates the individual, household, and societal interventions implemented during the Covid-19 pandemic and will be used to review and update relevant national guidelines accordingly, as recommended by the WHO [20]. Despite the European Commission intention “to continue to analyze the proportionality of measures taken by Member States to deal with the COVID-19 pandemic as the situation evolves and to intervene to request the lifting of measures considered disproportionate, especially when they have an impact on the Single Market.” [41], to our knowledge, the EU Commission has not intervened yet, although some measures’ perceived restrictiveness greatly outweighed the perceived effectiveness, as found in this study. Future studies should investigate the negative social, financial, and psychological effects of each containment measure in order to inform the debate about how to respond to pandemics without violating the proportionality principle.

## 5. Conclusions

This study provides unique data on which measures are perceived by citizens as the most effective and most restrictive to their human rights and which measures require balancing between the negative financial, psychological, and social effects and the desired outcome in order to protect the rule of law. Policymakers should implement the least restrictive and most effective public health measures first during pandemic emergencies. Further, governments that suffer low levels of trust should put more effort into persuading citizens, especially men, in the effectiveness of the proposed measures. They can achieve the desired outcome to stop the spread of the virus by increasing citizens’ compliance with the measures instead of implementing a combination of many restrictive measures, which has the opposite effect on citizens’ adherence. Policymakers should refrain from applying measures that are perceived as more restrictive for citizens’ human rights than effective and lacking objective evidence on the effectiveness of delaying the transmission of the virus. Public education campaigns should utilize evidence-based information gathered during the Covid-19 pandemic in order to maximize compliance with these measures.

## Figures and Tables

**Table 1:** Compliance with the five most relevant measures for citizens’ containment.

% People Affected by a Measure	Average Compliance (Lowest, Highest per Country ^1^, %)	Bivariate Associations: Factors Making People More (↑) or Less (↓) Compliant with the Measure	Multivariable Associations: Significant Factors Improving (↑) or Reducing (↓) Compliance When Factor Increased by 10%	Message for Policymakers
78% affected by “Keep at least 1–2 m away from other people (social distancing)”	73 (NL 53, IN 86)	Perceived restrictiveness (↓) Perceived effectiveness (↑) Following more pandemic news (↑) Older (↑) Higher education (↑) Trusting hospitals and government more (↑) More extreme government response (↓) More truthful government (↑) More stressed about the pandemic (↑) More afraid of getting infected (↑) Female gender (↑) Health conditions, comorbidities (↑) Medical staff (↑) Covid-19 infection in the family (↑) Job Loss without payment (↓)	Fear of getting infected (↑ 10.5%) Stressed by the outbreak (↑ 5%) Perceived effectiveness (↑ 5%) Trust in government (↑ 4%) Females (↑ 3.5%) than males Older compared to person 1 year younger (↑ 1%) More extreme government response (↓ 6%) Higher level of restrictiveness (↓ 1%)	To increase the rate of compliance with social distancing, policymakers should focus their efforts on increasing trust in Government, avoid extreme governmental policies, and convince citizens that social distancing is an effective strategy for preventing the infection, targeting mainly male and younger citizens.
73% affected by “Cancellation of all mass gatherings and events (cultural, sport, scientific or religious)”	81 (NL 68, IN 90)	Perceived restrictiveness (↓) Perceived effectiveness (↑) Following more pandemic news (↑) Older (↑) Higher education (↑) Trusting hospitals and government more (↑) More extreme government response (↓) More truthful government (↑) More stressed about the pandemic (↑) More afraid of getting infected (↑) Female gender (↑) Health conditions, comorbidities (↑) Medical staff (↑) Covid-19 infection in the family (↑) Job Loss without payment (↓)	Fear of getting infected (↑ 9.5%) Stressed by the outbreak (↑ 7%) Trust in government (↑ 7%) Perceived effectiveness (↑ 4%) Females (↑ 3.4%) than males Higher education (↑ 1.1%) Perceived restrictiveness (↑ 0.2%) More extreme government response (↓ 6%)	To increase the rate of compliance with not attending mass gatherings, policymakers should focus their efforts on distributing more information of how mass gatherings have the potential to amplify disease transmission, on increasing trust in the national Government, and avoiding extreme reactions by the Government.
66% affected by “Recommendation on washing hands more frequently”	87 (NL 65, RO 92)	Perceived restrictiveness (↓) Perceived effectiveness (↑) Following more pandemic news (↑) Older (↑) Higher education (↑) Trusting hospitals and government more (↑) More extreme government response (↓) More truthful government (↑) More stressed about the pandemic (↑) More afraid of getting infected (↑) Female gender (↑) Health conditions, comorbidities (↑) Medical staff (↑) Covid-19 infection in the family (↑) No Job Loss (↑)	Fear of getting infected (↑ 11%) Stressed by the outbreak (↑ 5%) Trust in government (↑ 5%) Perceived effectiveness (↑ 3%) Females (↑ 2.2%) than males With risky health conditions (↑ 1.2%) than no conditions More extreme government response (↓ 7%) Higher level of restrictiveness (↓ 1%)	To increase the rate of compliance with washing hands, the policymakers should focus their efforts on distributing more information of how effective this measure is in stopping the transmission of Covid-19 infection, to increase trust in the Government and avoid extreme government responses and the implementation of highly restrictive containment measures.
65% affected by “Keeping respiratory hygiene by mandatory wearing of a mask in public places”	73 (NL 41, IN 89)	Perceived restrictiveness (↓) Perceived effectiveness (↑) Following more pandemic news (↑) Younger (↑) Higher education (↑) Trusting hospitals and government more (↑) More extreme government response (↓) More truthful government (↑) More stressed about the pandemic (↑) More afraid of getting infected (↑) Female gender (↑) Health conditions, comorbidities (↑) Medical staff (↑) Covid-19 infection in the family (↑) No Job Loss (↑)	Fear of getting infected (↑ 11.5%) Trust in government (↑ 11%) Perceived effectiveness (↑ 4%) Females (↑ 4.8%) than males Stressed by the outbreak (↑ 2%) More extreme government response (↓ 13%) Higher level of restrictiveness (↓ 1%)	To increase the rate of compliance with wearing masks, the policymakers should focus their efforts on the following: increase public awareness of the health risks related to Covid-19 infections, increase the trust in the Government, avoid extreme reaction of the Government and highly restrictive measures, and provide evidence to citizens that masks are effective in cutting down transmission of the SARS-CoV-2 virus.
59% affected by “Mandatory stay-at-home, except for essential journeys”	76 (NL 64, IN 90)	Perceived restrictiveness (↓) Perceived effectiveness (↑) Following more pandemic news (↑) Older (↑) Higher education (↑) Trusting hospitals and government more (↑) More extreme government response (↓) More truthful government (↑) More stressed about the pandemic (↑) More afraid of getting infected (↑) Female gender (↑) Health conditions, comorbidities (↑) Medical staff (↑) Covid-19 infection in the family (↑) Job Loss without payment (↓)	Fear of getting infected (↑ 11.5%) Stressed by the outbreak (↑ 7%) Trust in government (↑ 7%) Higher education (↑ 7%) Perceived effectiveness (↑ 4%) Females (↑ 4.9%) than males Nonessential staff (↑ 2.4%) than medical and other essential staff Older compared to person 1 year younger (↑ 1%) More extreme government response (↓ 6%)	To increase the rate of compliance with the mandatory stay-at-home requirement, the policymakers should focus their efforts on distributing more information of how effective this measure is in stopping the transmission of the virus, targeting mainly male, younger, and lower-educated citizens. They should also increase trust in the Government and avoid extreme governmental responses.

^1^ ISO codes for the representation of names of countries: Belgium (BE), Netherlands (NL), Bulgaria (BG), Czech Republic (CZ), Finland (FI), India (IN), Latvia (LV), Poland (PL), Romania (RO), and Sweden (SE).

## Data Availability

The data presented in this study will be available after data anonymization at https://zenodo.org/ (accessed on 1 January 2021) when all data from this international study are analyzed and reported/published. The data are not currently publicly available due to privacy restrictions.

## Supplementary Materials

The following are available online at https://www.mdpi.com/1660-4601/18/7/3806/s1, Table S1: Type and number of containment measures implemented by countries, marked with “X”, Table S2: Survey questions reported in this study, Table S3: Demographics and variations across countries (%, mean), Table S4: Effectiveness, restrictiveness, and compliance per country, Table S5: Bivariate correlations with compliance (Spearman rho), Table S6: Other factors for compliance, Table S7: Multivariable model factors for compliance (stepwise selection, controlling for country effect, only significant associations (*p* < 0.05) are reported in the Table).
