# Supplementary material for: Perceived Effectiveness, Restrictiveness, and Compliance with Containment Measures against the Covid-19 Pandemic: An International Comparative Study in 11 Countries
Source: Int J Environ Res Public Health. 2021 Apr 6;18(7):3806. doi: 10.3390/ijerph18073806 (PMC8038651; doi:10.3390/ijerph18073806)
Supplement: Supplementary file 1 [file ijerph-18-03806-s001.pdf]

**Table S1. Type and number of containment measures implemented by countries, marked with 'X'**

| Type of containment measure | England | Nether-lands | Finland | Sweden | Bulgaria | Romania | Poland | Czech Republic | Latvia | India |
|---|---|---|---|---|---|---|---|---|---|---|
| ***Hygienic and health measures*** | | | | | | | | | | |
| 1. Recommendation on washing hands more frequently | X | X | X | X | X | X | X | X | X | X |
| 2. Keeping respiratory hygiene by mandatory wearing a mask in public places | | | | | X | X | X | X | | X |
| 3. Mandatory testing of all suspected cases | | | | | | | X | | | |
| 4. Recommendation on coughing or sneezing into your elbow | X | X | X | X | X | X | X | | X | |
| 5. Recommendation on NOT shaking hands | X | X | X | X | X | X | X | X | X | X |
| 6. Mandatory reporting symptoms of illness to health authorities | | X | | | | | X | | X | X |
| 7. Enhanced cleaning and disinfection procedures | | X | X | X | X | X | X | X | X | |
| ***Social-distancing measures*** | | | | | | | | | | |
| 8. Cancellation of all mass gatherings and events (cultural, sport, scientific or religious) | X | X | X | X | X | X | X | X | X | X |
| 9. Funerals and weddings are forbidden or the number of attendees is restricted | X | X | X | X | X | X | X | X | X | >20 |
| 10. Closure of preschools, nurseries and primary schools | X | X | | | X | X | X | X | X | X |
| 11. Closure of secondary schools, colleges and universities | X | X | X | X | X | X | X | X | X | X |
| 12. Final exams are cancelled or postponed | X | | | | X | | X | | X | X |
| 13. Recommendation on not using public transportation (e.g., bus, trains, ships, etc.) | X | X | | X | X | X | | X | X | X |
| 14. Recommendation on working from home if possible | X | X | X | X | X | X | X | | X | X |
| 15. Keep at least 1-2 meters away from other people | X | X | X | X | X | X | X | X | X | X |
| 16. Mandatory stay-at-home, unless it is absolutely essential to leave the home | X | | | | X | X | X | X | | X |
| 17. Introducing market / shopping hours for vulnerable groups | X | | | X | X | X | X | | | Given at door steps free of cost |
| 18. A ban on visiting national and nature parks and mountains | X | | | | X | X | X | | | X |
| 19. Dissolution of the Parliament - National Assembly meets only on bills and acts related to the state of emergency | Virtual meetings | | | | X | | | | | X |
| 20. Closure of pubs, cafes, restaurants, except for delivery and takeaway services. | X | X | X | | X | X | X | X | | X |
| 21. Closure of cinemas, theatres, opera, concert venues, libraries, museums, heritage sites, discos, gambling halls, sport indoor and outdoor facilities. | X | X | X | | X | X | X | X | X | X |
| 22. Closure of non-essential shops and large shopping malls. | X | | | | X | X | | X | | X |
| 23. Limited number of citizens are allowed to gather and visit open and closed public places. | X | | | | X | More than 3 | X | | X | |
| 24. Closure of playgrounds | X | X | | | X | X | X | X | X | X |
| 25. Closure of all hotels and tourist accommodation | X | | | | X | X | | X | | X |
| 26. Closure of the judiciary and courts | | | | | X | | X | | X | Virtual meetings |
| 27. Discontinued are all elective medical surgeries and procedures | X | X | | X | X | X | X | X | X | X |

| Type of containment measure | England | Nether-lands | Finland | Sweden | Bulgaria | Romania | Poland | Czech Republic | Latvia | India |
|---|---|---|---|---|---|---|---|---|---|---|
| 28. Visits to housing services for the elderly and other at-risk groups are prohibited | | X | X | X | | X | | | X | |
| 29. A curfew order | | | | | | X | | | | X |
| 30. People are not allowed to receive guests in their home, or the number of guests is restricted | | X | | | | X | | X | X | X |
| ***Isolation of symptomatic individuals and quarantine*** | | | | | | | | | | |
| 31. Isolation of ill persons at home for certain period of time | X | X | X | | X | X | X | X | X | |
| 32. Obligatory admission to a hospital of seriously ill persons | | | | | X | X | X | X | | |
| 33. Placement in mandatory home quarantine of contacts | | X | X | | X | X | X | | X | X |
| 34. Placement in mandatory home quarantine of citizens returning from abroad | | | X | | X | X | X | X | X | X |
| ***Travel restrictions and border controls*** | | | | | | | | | | |
| 35. Placement of cordon sanitaire on areas infected by a disease such as big cities (i.e. a guarded line preventing anyone from leaving the infected area) | | | | | X | X | | | | X |
| 36. Using screening procedures such as asking travelers if they have symptoms of influenza; have had close contact with someone with influenza; performing a visual screen for signs of influenza or measuring body temperature | | X | X | | X | X | X | X | X | X |
| 37. Border closures: partial or total closure of a land border (e.g., to people from outside the EU) | | X | X | | X | X | X | X | X | X |
| 38. Flight suspensions: government bans on flights from or to the country | | X | | | X | X | X | X | X | X |
| 39. Present a health screening form when entering the country | | X | | | | X | | X | X | X |
| 40. Citizens are not allowed to leave the country | | | X | | | | X | | | X |
| ***Surveillance measures*** | | | | | | | | | | |
| 41. Contact tracing assessment of Covid-19 transmission | | X | X | | X | | X | X | | |
| 42. Police forces are allowed to request and obtain citizens' personal information from internet and telephone providers | | | | | X | | | | X | |
| 43. Mass testing for Covid-19 (i.e., expanded targeted testing or random testing of the general population, irrespective of symptoms) | | X | X | | | | | | | |
| ***Other measures*** | | | | | | | | | | |
| 44. Declaring state of emergency in the country | | | X | | X | X | | X | X | X |
| 45. Penalties or fines for non-compliance with covid-19 containment measures | X | X | X | | X | X | X | X | X | X |
| **Total number of measures: 45** | **24** | **27** | **22** | **13** | **37** | **36** | **33** | **28** | **30** | **35** |

**Table S2. Survey questions reported in this study**

| Question | Answer |
|---|---|
| 1.What is your gender? | A. Female<br>B. Male<br>C. Other |
| 2.Which year were you born? | |
| 3.What is the highest level of schooling you have completed or the highest degree you have received? | A. Primary school<br>B. High school<br>C. Professional qualification (not university)<br>D. College degree E) Bachelor degree<br>E. Masters degree<br>F. Doctoral degree |
| 4.Did you belong to the group of essential staff (e.g. medical staff, food preparation & serving workers, stock clerks and order fillers, etc.) or nonessential workers during the coronavirus outbreak? | A. Medical staff<br>B. Other essential staff<br>C. Nonessential staff |
| Have you lost your job or income due to the containment measures taken by your national government? | A. No, I didn't<br>B. I lost my job<br>C. My working hours were reduced<br>D. I had to take unpaid leave<br>E. I was furloughed |
| 5.Have you gotten a COVID-19 Pandemic Unemployment Payment or other types of financial compensation? | A. Yes<br>B. No |
| Have you been infected and tested positive for the coronavirus? | A. No<br>B. I have experienced COVID-19 symptoms, but I have not been tested<br>C. I have experienced COVID-19 symptoms and have been tested positive<br>D. I have been tested positive, but I have not experienced any COVID-19 symptoms |
| 6.Have any of your family members or close friends been infected with the coronavirus? | A. No<br>B. Yes<br>C. Possibly |
| 7.Are you having any of the following health conditions: cardiovascular diseases, diabetes, hepatitis B, chronic obstructive pulmonary disease, chronic kidney disease, liver disease, cancer, morbid obesity, and hypertension? | A. Yes<br>B. No |
| 8.How many hours per day on average have you been engaged in following the news related to the coronavirus outbreak on TV, radio, newspapers or social media? | |
| 9.How much do you trust that the hospitals in your country have the resources and expertise to provide the best treatment available to people infected with the coronavirus? | Measured on 11-point Likert scale from 0 (Strongly distrust) to 10 (Strongly trust) |
| 10.How much do you trust your national government to take care of its citizens? | Measured on 11-point Likert scale from 0 (Strongly distrust) to 10 (Strongly trust) |
| 11.Do you think the reaction of your national government to the coronavirus outbreak has been appropriate, too extreme or not sufficient? | Measured on 11-point Likert scale from 0 (Not at all sufficient), 5 (Reaction is appropriate) to 10 (Extremely stressful) |
| 12.How factually truthful do you think your national government has been about the coronavirus outbreak? | Measured on 11-point Likert scale from 0 (Very untruthful) to 10 (Very truthful) |
| 13.How stressful have you found the crisis with the coronavirus outbreak? | Measured on 11-point Likert scale from 0 (Not at all stressful) to 10 (Extremely stressful) |
| 14.During the past week were there any social situations (e.g. work, public transportation, movie, theaters, crowds, shopping malls) you avoided, or felt afraid of (uncomfortable in, wanted to avoid or leave), because of fear of getting infected by the coronavirus? Are there any other situations that you would have avoided or been afraid of if they had come up during the past week, for the same reason? Please rate your level of fear and avoidance this past week. | 1-None: no fear or avoidance; 2-Mild: occasional fear and/or avoidance but I could usually confront or endure the situation. There was little or no modification of my lifestyle due to this; 3-Moderate: noticeable fear and/or avoidance but still manageable. I avoided some situations, but I could confront them with a companion. There was some modification of my lifestyle because of this, but my overall functioning was not impaired; 4-Severe: extensive avoidance. Substantial modification of my lifestyle was required to accommodate the avoidance making it difficult to manage usual activities; 5-Extreme: pervasive disabling fear and/or avoidance. Extensive modification in my lifestyle was required such that important tasks were not performed. |
| 15.Do you think that the measures to prevent the spread of COVID-19 virus have more negative implications than Covid-19 itself ? | 1-Strongly disagree, 2- Disagree, 3-Neither agree or disagree, 4- Agree, 5- Strongly agree |
| 16.Has this measure been applied in your country? | A. Yes, this measure has been applied in my country and affected me personally -> Go to Question 17 & 18<br>B. Yes, this measure has been applied in my country, but did not affect me personally -> Go to Question 19<br>C. No, this measure has not been applied in my country -> Go to next measure<br>D. I don't know -> Go to next measure |
| 17.To what extent have you followed this measure? Please try to be as honest as possible. Your answers will be kept confidential. | Measured on 11-point Likert scale from 0 (Not at all) to 10 (Strictly every day) |
| 18.To what extent has this measure caused you any discomfort or restricted your personal freedom and your fundamental human rights to engage in work, education, meet other people, move freely within the country or to visit other countries? | Measured on 11-point Likert scale from 0 (Not at all restrictive) to 10 (Extremely restrictive) |
| 19.How effective do you find this measure to prevent the spread of COVID-19 virus? | Measured on 11-point Likert scale from 0 (Not at all effective) to 10 (Extremely effective) |

**Table S3. Demographics & variations across countries (%, mean)**

| Characteristics / Variables | UK N=653 | Belgium N=374 | Netherlands N=864 | Bulgaria N=1868 | Czech Rep N=723 | Finland N=542 | India N=779 | Latvia N=643 | Poland N=1008 | Romania N=1504 | Sweden N=585 | Total N=9543 |
|---|---|---|---|---|---|---|---|---|---|---|---|---|
| Female (%) | 59.0 | 75.1 | 68.2 | 80.3 | 85.6 | 83.2 | 31.2 | 85.5 | 78.8 | 63.2 | 77.9 | 71.4 |
| Age (mean) | 57.0 | 52.6 | 55.1 | 43.4 | 41.0 | 50.3 | 47.6 | 45.3 | 38.1 | 50.5 | 51.9 | 47.5 |
| Higher Education (BA, MA, PhD) (%) | 42.0 | 51.9 | 15.5 | 79.7 | 34.4 | 26.8 | 82.3 | 70.6 | 69.0 | 71.8 | 63.2 | 60.0 |
| Medical Staff (%) | 7.7 | 9.9 | 12.0 | 4.3 | 31.4 | 15.1 | 28.8 | 11.2 | 4.4 | 5.1 | 16.4 | 11.5 |
| Other Essential Staff (%) | 21.9 | 15.8 | 18.8 | 6.2 | 10.8 | 10.1 | 14.2 | 19.3 | 11.3 | 7.5 | 20.3 | 12.5 |
| Non-essential Staff (%) | 70.4 | 74.3 | 69.2 | 89.5 | 57.8 | 74.7 | 57.0 | 69.5 | 84.3 | 87.4 | 63.2 | 76.0 |
| Lost Job (%) | 23.0 | 13.6 | 16.2 | 35.1 | 23.5 | 12.9 | 38.5 | 25.5 | 36.5 | 24.1 | 8.4 | 26 |
| Received Covid-19 Unemployment Compensation of Lost Job (%) | 35.3 | 54.9 | 29.3 | 10.4 | 25.3 | 37.1 | 9.0 | 18.9 | 16.8 | 14.6 | 26.5 | 17.9 |
| No Covid-19 symptoms & not tested positive (%) | 83.3 | 89.0 | 84.3 | 92.9 | 91.8 | 92.3 | 95.8 | 95.3 | 95.4 | 96.8 | 76.8 | 91.5 |
| Family Not infected with Covid-19 (%) | 67.4 | 63.4 | 70.6 | 83.6 | 85.9 | 86.5 | 74.5 | 90.0 | 86.3 | 81.4 | 45.8 | 78.2 |
| With Health Conditions (%) | 30.3 | 28.1 | 32.6 | 23.2 | 27.5 | 37.6 | 31.1 | 32.3 | 28.5 | 36.8 | 34.9 | 30.5 |
| Time News (mean in hours) | 1.7 | 1.7 | 2.1 | 1.9 | 1.8 | 1.3 | 2 | 1.2 | 1.5 | 2.2 | 1.5 | 1.8 |
| Trust Hospitals (mean): measured on 11-point Likert scale from 0 (Strongly distrust) to 10 (Strongly trust) | 5.9 | 6.8 | 5.7 | 3.3 | 6.6 | 7.8 | 5.2 | 5.3 | 2.9 | 3 | 6.7 | 4.7 |
| Trust Government (mean): measured on 11-point Likert scale from 0 (Strongly distrust) to 10 (Strongly trust) | 3 | 3.6 | 3 | 2 | 3 | 6.6 | 5.7 | 3.9 | 1.3 | 3 | 6 | 3.3 |
| Reaction Government (mean): measured on 11-point Likert scale from 0 (Not at all sufficient), 5 (Reaction is appropriate) to 10 (Extremely stressful) | 5 | 5.8 | 7 | 5.8 | 6 | 5 | 5 | 6.8 | 6.2 | 5.1 | 3.9 | 5.7 |
| Truthful Government (mean): measured on 11-point Likert scale from 0 (Very untruthful) to 10 (Very truthful) | 2.6 | 6 | 6.9 | 3.2 | 4 | 6.3 | 5.5 | 4.8 | 1.7 | 3.6 | 6.4 | 4.2 |
| Stress Outbreak (mean): measured on 11-point Likert scale from 0 (Not at all stressful) to 10 (Extremely stressful) | 6.2 | 6.6 | 6 | 6.8 | 5.6 | 6.2 | 6.4 | 5.6 | 6 | 7.7 | 6 | 6.5 |
| Fear of Infection (mean): see Table 2, question 14 | 1.9 | 2 | 1.6 | 2 | 1.6 | 2 | 2.3 | 1.7 | 1.9 | 2 | 2.5 | 1.9 |
| More negative implications (mean): measured on 5-point Likert scale from 1 (Strongly disagree) to 5 (Strongly agree) | 3.5 | 3.1 | 3.9 | 3.3 | 3.5 | 2.3 | 3.0 | 2.7 | 3.4 | 2.9 | 2.5 | 3.1 |

## Table S4. Effectiveness, Restrictiveness, Compliance per country

| Measure (% affected people)[1] /Variables N (%) | UK | Belgium | Netherlands | Bulgaria | Czech Rep | Finland | India | Latvia | Poland | Romania | Sweden | Mean total |
|---|---|---|---|---|---|---|---|---|---|---|---|---|
| **1. Keep at least 1-2 meters away from other people (78%)** | | | | | | | | | | | | |
| Effectiveness N=6943 (%) | 57 | 68 | 35 | 66 | 60 | 76 | 85 | 62 | 51 | 69 | 82 | 64 |
| Restrictiveness N=5454 (%) | 62 | 62 | 81 | 49 | 60 | 40 | 54 | 49 | 55 | 60 | 44 | 56 |
| Compliance N=5458 (%) | 72 | 78 | 53 | 75 | 70 | 81 | 86 | 75 | 65 | 74 | 83 | 73 |
| **2. Cancellation of all mass gatherings and events (cultural, sport, scientific or religious) (73%)** | | | | | | | | | | | | |
| Effectiveness N=6895 (%) | 59 | 64 | 47 | 66 | 68 | 85 | 86 | 63 | 59 | 66 | 84 | 67 |
| Restrictiveness N=5138 (%) | 70 | 78 | 82 | 70 | 79 | 50 | 56 | 66 | 71 | 76 | 51 | 69 |
| Compliance N=5136 (%) | 80 | 85 | 68 | 82 | 81 | 89 | 90 | 80 | 75 | 79 | 89 | 81 |
| **3. Recommendation on washing hands more frequently (66%)** | | | | | | | | | | | | |
| Effectiveness N=6917 (%) | 60 | 71 | 45 | 72 | 68 | 82 | 77 | 65 | 62 | 76 | 78 | 69 |
| Restrictiveness N=4644 (%) | 16 | 20 | 33 | 10 | 18 | 13 | 38 | 17 | 13 | 17 | 13 | 17 |
| Compliance N=4635 (%) | 82 | 84 | 65 | 90 | 88 | 90 | 87 | 89 | 85 | 92 | 91 | 87 |
| **4. Keeping respiratory hygiene by mandatory wearing of a mask in public places (65%)** | | | | | | | | | | | | |
| Effectiveness N=5744 (%) | 39 | 51 | 20 | 53 | 57 | 62 | 81 | 43 | 42 | 62 | 64 | 52 |
| Restrictiveness N=4549 (%) | 60 | 66 | 81 | 54 | 70 | 64 | 51 | 57 | 62 | 64 | n/a | 60 |
| Compliance N=4540 (%) | 61 | 76 | 41 | 78 | 79 | 74 | 89 | 62 | 68 | 77 | n/a | 73 |
| **5. Mandatory stay-at-home, except for essential journeys (59%)** | | | | | | | | | | | | |
| Effectiveness N=5637 (%) | 55 | 58 | 40 | 48 | 60 | 78 | 85 | 64 | 47 | 59 | 83 | 58 |
| Restrictiveness N=4105 (%) | 74 | 79 | 84 | 78 | 81 | 54 | 64 | 71 | 80 | 79 | 58 | 76 |
| Compliance N=4110 (%) | 77 | 82 | 64 | 74 | 73 | 83 | 90 | 74 | 68 | 79 | 88 | 76 |
| **6. Recommendation on NOT shaking hands (59%)** | | | | | | | | | | | | |
| Effectiveness N=6387 (%) | 53 | 73 | 43 | 56 | 58 | 77 | 83 | 56 | 46 | 63 | 77 | 60 |
| Restrictiveness N=4148 (%) | 32 | 39 | 58 | 34 | 41 | 17 | 38 | 28 | 36 | 45 | 17 | 36 |
| Compliance N=4147 (%) | 81 | 90 | 68 | 71 | 74 | 93 | 92 | 78 | 65 | 70 | 92 | 77 |
| **7. Closure of pubs, cafes, restaurants, except for delivery and takeaway services (58%)** | | | | | | | | | | | | |
| Effectiveness N=6450 (%) | 55 | 59 | 38 | 60 | 58 | 79 | 84 | 53 | 46 | 59 | 80 | 59 |
| Restrictiveness N=4089 (%) | 72 | 70 | 79 | 65 | 78 | 46 | 59 | 69 | 71 | 76 | 50 | 69 |
| Compliance N=4088 (%) | 83 | 87 | 71 | 90 | 86 | 90 | 92 | 77 | 82 | 87 | 76 | 85 |
| **8. Closure of cinemas, theaters, opera, concert venues, libraries, museums, heritage sites, discos and/or gambling halls (56%)** | | | | | | | | | | | | |
| Effectiveness N=6725 (%) | 56 | 60 | 42 | 60 | 61 | 78 | 88 | 59 | 49 | 61 | 78 | 61 |
| Restrictiveness N=3947 (%) | 71 | 73 | 79 | 70 | 77 | 52 | 59 | 72 | 73 | 77 | 58 | 71 |
| Compliance N=3956 (%) | 88 | 90 | 75 | 92 | 90 | 91 | 94 | 85 | 85 | 90 | 92 | 89 |
| **9. Enhanced cleaning and disinfection procedures (55%)** | | | | | | | | | | | | |
| Effectiveness N=6419 (%) | 59 | 66 | 37 | 71 | 70 | 77 | 83 | 68 | 61 | 75 | 83 | 68 |
| Restrictiveness N=3855 (%) | 28 | 39 | 54 | 34 | 44 | 23 | 43 | 30 | 35 | 37 | 20 | 36 |
| Compliance N=3853 (%) | 73 | 75 | 50 | 78 | 79 | 81 | 85 | 77 | 72 | 80 | 87 | 76 |
| **10. Limited number of citizens are allowed to visit open and closed public places, and/or to gather at home (54%)** | | | | | | | | | | | | |
| Effectiveness N=6235 (%) | 50 | 56 | 35 | 53 | 53 | 76 | 82 | 55 | 42 | 59 | 81 | 57 |
| Restrictiveness N=3765 (%) | 75 | 78 | 85 | 72 | 77 | 51 | 61 | 69 | 78 | 79 | 49 | 73 |
| Compliance N=3762 (%) | 76 | 79 | 52 | 76 | 72 | 86 | 90 | 77 | 65 | 80 | 88 | 74 |
| **11. Recommendation on coughing or sneezing into your elbow (50%)** | | | | | | | | | | | | |
| Effectiveness N=6274 (%) | 52 | 60 | 50 | 58 | 58 | 75 | 79 | 53 | 50 | 68 | 74 | 61 |
| Restrictiveness N=3491 (%) | 13 | 18 | 24 | 12 | 19 | 11 | 39 | 15 | 12 | 23 | n/a | 17 |
| Compliance N= 3486 (%) | 73 | 75 | 72 | 77 | 76 | 90 | 84 | 80 | 75 | 81 | 90 | 79 |
| **12. Recommendation on working from home if possible (48%)** | | | | | | | | | | | | |
| Effectiveness N=6909 (%) | 58 | 68 | 46 | 66 | 64 | 79 | 84 | 64 | 56 | 67 | 81 | 66 |
| Restrictiveness N=3400 (%) | 55 | 54 | 73 | 51 | 51 | 39 | 55 | 48 | 54 | 57 | 37 | 53 |

| Measure (% affected people)[1] /Variables N (%) | UK | Belgium | Netherlands | Bulgaria | Czech Rep | Finland | India | Latvia | Poland | Romania | Sweden | Mean total |
|---|---|---|---|---|---|---|---|---|---|---|---|---|
| Compliance N=3397 (%) | 75 | 82 | 67 | 86 | 77 | 83 | 85 | 82 | 83 | 83 | 80 | 82 |
| 13. **Declaring state of emergency in the country (48%)** | | | | | | | | | | | | |
| Effectiveness N=4329 (%) | 42 | 53 | 23 | 48 | 53 | 75 | 80 | 59 | 47 | 58 | 93 | 55 |
| Restrictiveness N=3334 (%) | 84 | 74 | 90 | 77 | 81 | 59 | 67 | 72 | 77 | 80 | n/a | 77 |
| Compliance N=3339 (%) | 72 | 82 | 48 | 84 | 81 | 88 | 88 | 84 | 76 | 88 | 100 | 83 |
| 14. **Closure of non-essential shops and/or large shopping malls (46%)** | | | | | | | | | | | | |
| Effectiveness N=5618 (%) | 53 | 53 | 34 | 56 | 60 | 73 | 84 | 56 | 46 | 60 | 86 | 57 |
| Restrictiveness N=3217 (%) | 67 | 69 | 81 | 63 | 71 | 49 | 58 | 63 | 69 | 72 | 68 | 67 |
| Compliance N=3221 (%) | 82 | 89 | 65 | 88 | 89 | 74 | 92 | 81 | 81 | 87 | 92 | 85 |
| 15. **A ban on visiting national and nature parks (46%)** | | | | | | | | | | | | |
| Effectiveness N=5215 (%) | 38 | 38 | 22 | 22 | 36 | 57 | 82 | 32 | 14 | 41 | 69 | 34 |
| Restrictiveness N=3204 (%) | 71 | 70 | 80 | 83 | 81 | 58 | 59 | 72 | 87 | 84 | 34 | 80 |
| Compliance N=3202 (%) | 76 | 78 | 45 | 76 | 66 | 58 | 91 | 67 | 64 | 79 | 76 | 74 |
| 16. **Recommendation on not using public transport (e.g bus, trains, ships, etc.) (42%)** | | | | | | | | | | | | |
| Effectiveness N=5670 (%) | 56 | 62 | 42 | 62 | 63 | 78 | 84 | 59 | 50 | 65 | 78 | 62 |
| Restrictiveness N=2976 (%) | 65 | 62 | 76 | 67 | 69 | 49 | 63 | 68 | 69 | 70 | 53 | 66 |
| Compliance N=2979 (%) | 76 | 77 | 68 | 72 | 72 | 80 | 91 | 68 | 68 | 76 | 79 | 75 |
| 17. **Closure of preschools, nurseries and/or primary schools (42%)** | | | | | | | | | | | | |
| Effectiveness N=6463 (%) | 46 | 44 | 30 | 60 | 56 | 73 | 88 | 56 | 51 | 64 | 68 | 57 |
| Restrictiveness N=2966 (%) | 49 | 73 | 76 | 67 | 77 | 53 | 53 | 75 | 74 | 70 | 36 | 67 |
| Compliance N=2958 (%) | 77 | 84 | 74 | 93 | 91 | 86 | 93 | 89 | 83 | 89 | 82 | 88 |
| 18. **Closure of secondary schools, colleges and/or universities (41%)** | | | | | | | | | | | | |
| Effectiveness N=6893 (%) | 51 | 53 | 36 | 61 | 57 | 77 | 86 | 57 | 52 | 64 | 69 | 60 |
| Restrictiveness N=2866 (%) | 53 | 68 | 69 | 63 | 72 | 47 | 54 | 70 | 67 | 69 | 40 | 63 |
| Compliance N=2867 (%) | 78 | 87 | 75 | 91 | 92 | 88 | 90 | 90 | 83 | 88 | 89 | 88 |
| 19. **Funerals and weddings are forbidden or the number of attendees is restricted (38%)** | | | | | | | | | | | | |
| Effectiveness N=6597 (%) | 53 | 60 | 42 | 61 | 59 | 76 | 86 | 52 | 56 | 66 | 79 | 62 |
| Restrictiveness N=2682 (%) | 58 | 65 | 76 | 54 | 67 | 39 | 52 | 58 | 65 | 70 | 41 | 60 |
| Compliance N=2675 (%) | 75 | 84 | 65 | 81 | 81 | 86 | 91 | 70 | 68 | 79 | 87 | 79 |
| 20. **Closure of indoor and/or outdoor sport facilities (38%)** | | | | | | | | | | | | |
| Effectiveness N=6397 (%) | 54 | 55 | 37 | 56 | 53 | 76 | 83 | 51 | 47 | 57 | 75 | 56 |
| Restrictiveness N=2632 (%) | 72 | 77 | 84 | 72 | 79 | 59 | 58 | 77 | 73 | 77 | 60 | 73 |
| Compliance N=2632 (%) | 86 | 87 | 75 | 88 | 86 | 88 | 91 | 83 | 78 | 86 | 83 | 85 |
| 21. **Introducing market / shopping hours for vulnerable groups (37%)** | | | | | | | | | | | | |
| Effectiveness N=6363 (%) | 47 | 50 | 36 | 48 | 37 | 70 | 82 | 51 | 28 | 54 | 66 | 50 |
| Restrictiveness N=2620 (%) | 45 | 42 | 44 | 55 | 72 | 41 | 60 | 51 | 70 | 74 | 43 | 60 |
| Compliance N=2610 (%) | 68 | 79 | 49 | 88 | 86 | 76 | 88 | 59 | 78 | 82 | 78 | 81 |
| 22. **Stopping of all elective medical surgeries and procedures (30%)** | | | | | | | | | | | | |
| Effectiveness N=6070 (%) | 42 | 38 | 24 | 44 | 42 | 57 | 74 | 39 | 27 | 35 | 53 | 41 |
| Restrictiveness N=2086 (%) | 80 | 68 | 83 | 75 | 80 | 59 | 62 | 85 | 90 | 85 | 62 | 78 |
| Compliance N=2082 (%) | 87 | 88 | 70 | 87 | 88 | 86 | 88 | 84 | 76 | 87 | 85 | 84 |
| 23. **Closure of hotels and tourist accommodation (30%)** | | | | | | | | | | | | |
| Effectiveness N=5814 (%) | 54 | 52 | 32 | 55 | 57 | 74 | 84 | 54 | 43 | 55 | 72 | 55 |
| Restrictiveness N=2088 (%) | 78 | 67 | 84 | 76 | 81 | 60 | 60 | 75 | 80 | 83 | 58 | 76 |
| Compliance N=2085 (%) | 85 | 86 | 69 | 89 | 88 | 85 | 92 | 84 | 78 | 86 | 85 | 86 |
| 24. **Mandatory reporting of symptoms of illness to health authorities (27%)** | | | | | | | | | | | | |
| Effectiveness N=5034 (%) | 54 | 63 | 40 | 66 | 67 | 82 | 81 | 61 | 53 | 67 | 83 | 64 |
| Restrictiveness N=1866 (%) | 35 | 37 | 63 | 30 | 37 | 29 | 43 | 28 | 40 | 50 | 34 | 38 |
| Compliance N=1864 (%) | 65 | 66 | 42 | 74 | 73 | 82 | 86 | 67 | 51 | 62 | 78 | 69 |

| Measure (% affected people)[1] /Variables N (%) | UK | Belgium | Netherlands | Bulgaria | Czech Rep | Finland | India | Latvia | Poland | Romania | Sweden | Mean total |
|---|---|---|---|---|---|---|---|---|---|---|---|---|
| **25. Closure of playgrounds (25%)** | | | | | | | | | | | | |
| Effectiveness N=5584 (%) | 45 | 41 | 23 | 45 | 49 | 72 | 82 | 48 | 41 | 56 | 77 | 49 |
| Restrictiveness N=1739 (%) | 64 | 63 | 81 | 75 | 71 | 46 | 58 | 71 | 76 | 78 | n/a | 72 |
| Compliance N=1737 (%) | 72 | 85 | 47 | 82 | 83 | 74 | 90 | 79 | 74 | 84 | 100 | 80 |
| **26. Visits to care homes for the elderly and other at-risk groups are prohibited (21%)** | | | | | | | | | | | | |
| Effectiveness N=5998 (%) | 62 | 62 | 31 | 72 | 72 | 77 | 82 | 63 | 54 | 67 | 79 | 65 |
| Restrictiveness N=1444 (%) | 70 | 70 | 86 | 59 | 67 | 60 | 57 | 72 | 71 | 65 | 57 | 67 |
| Compliance N=1446 (%) | 82 | 89 | 66 | 90 | 94 | 92 | 89 | 88 | 74 | 88 | 91 | 85 |
| **27. Final exams are cancelled or postponed (20%)** | | | | | | | | | | | | |
| Effectiveness N=5865 (%) | 39 | 39 | 29 | 48 | 44 | 68 | 79 | 43 | 42 | 53 | 65 | 49 |
| Restrictiveness N=1425 (%) | 39 | 61 | 64 | 57 | 69 | 32 | 56 | 62 | 65 | 68 | 39 | 59 |
| Compliance N=1421 (%) | 66 | 85 | 62 | 88 | 85 | 75 | 87 | 89 | 78 | 81 | 88 | 82 |
| **28. Flight suspensions: government bans on flights from or to the country (20%)** | | | | | | | | | | | | |
| Effectiveness N=5205 (%) | 56 | 67 | 50 | 57 | 65 | 83 | 84 | 68 | 49 | 64 | 78 | 63 |
| Restrictiveness N=1411 (%) | 87 | 76 | 87 | 87 | 85 | 66 | 66 | 87 | 88 | 81 | 73 | 82 |
| Compliance N=1404 (%) | 82 | 89 | 81 | 94 | 94 | 87 | 91 | 94 | 84 | 90 | 93 | 90 |
| **29. Partial closure of a land border: e.g. people from outside the EU can't enter the country (18%)** | | | | | | | | | | | | |
| Effectiveness N=5347 (%) | 57 | 64 | 46 | 60 | 62 | 86 | 84 | 68 | 49 | 65 | 73 | 64 |
| Restrictiveness N=1235 (%) | 77 | 80 | 89 | 80 | 82 | 53 | 60 | 83 | 82 | 76 | 54 | 77 |
| Compliance N=1227 (%) | 88 | 83 | 69 | 91 | 90 | 90 | 89 | 90 | 79 | 86 | 95 | 87 |
| **30. Using screening procedures such as asking travelers if they have symptoms of flu, have been in contact with someone with flu, performing a visual screen for signs of flu or measuring body temperature (18%)** | | | | | | | | | | | | |
| Effectiveness N=4555 (%) | 52 | 59 | 34 | 53 | 57 | 77 | 82 | 61 | 43 | 54 | 67 | 55 |
| Restrictiveness N=1273 (%) | 55 | 54 | 72 | 49 | 55 | 33 | 49 | 52 | 52 | 58 | 40 | 54 |
| Compliance N=1278 (%) | 81 | 78 | 59 | 88 | 90 | 82 | 89 | 84 | 79 | 84 | 93 | 82 |
| **31. Recommendation to quarantine of citizens who belong to high risk groups or who have been in contact with infected people (17%)** | | | | | | | | | | | | |
| Effectiveness N=5867 (%) | 64 | 73 | 48 | 76 | 74 | 84 | 87 | 76 | 61 | 75 | 86 | 72 |
| Restrictiveness N=1187 (%) | 69 | 51 | 76 | 60 | 67 | 57 | 58 | 69 | 73 | 68 | 66 | 64 |
| Compliance N=1188 (%) | 78 | 69 | 62 | 87 | 89 | 88 | 90 | 85 | 73 | 81 | 86 | 82 |
| **32. Introducing a curfew order (17%)** | | | | | | | | | | | | |
| Effectiveness N=2204 (%) | 40 | 42 | 15 | 29 | 45 | 48 | 79 | 32 | 18 | 56 | 50 | 53 |
| Restrictiveness N=1195 (%) | 69 | 68 | 91 | 75 | 88 | 80 | 66 | 57 | 71 | 81 | n/a | 77 |
| Compliance N=1198 (%) | 69 | 83 | 46 | 70 | 66 | 5 | 90 | 100 | 67 | 84 | 100 | 81 |
| **33. Total closure of a land border: citizens are not allowed to leave the country (16%)** | | | | | | | | | | | | |
| Effectiveness N=3261 (%) | 53 | 58 | 33 | 51 | 61 | 83 | 84 | 65 | 45 | 60 | 82 | 60 |
| Restrictiveness N=1077 (%) | 87 | 78 | 84 | 86 | 85 | 80 | 63 | 87 | 86 | 85 | 68 | 82 |
| Compliance N=1075 (%) | 94 | 88 | 70 | 92 | 92 | 91 | 90 | 93 | 86 | 90 | 89 | 90 |
| **34. Placement of cordon sanitaire on areas with certain infection levels, such as big cities (i.e. a guarded line preventing anyone from leaving the infected area) (15%)** | | | | | | | | | | | | |
| Effectiveness N=3322 (%) | 51 | 55 | 28 | 48 | 70 | 78 | 84 | 69 | 34 | 68 | 70 | 61 |
| Restrictiveness N=1047 (%) | 58 | 80 | 55 | 85 | 46 | 54 | 57 | 90 | 66 | 68 | 40 | 74 |
| Compliance N=1049 (%) | 76 | 79 | 49 | 82 | 91 | 92 | 89 | 80 | 47 | 83 | 85 | 84 |
| **35. Mandatory isolation of ill persons for certain periods of time (14%)** | | | | | | | | | | | | |
| Effectiveness N=5964 (%) | 68 | 81 | 55 | 82 | 82 | 91 | 89 | 78 | 68 | 77 | 90 | 78 |
| Restrictiveness N=998 (%) | 54 | 63 | 79 | 50 | 64 | 48 | 60 | 57 | 67 | 70 | 49 | 60 |
| Compliance N=1000 (%) | 83 | 84 | 58 | 90 | 87 | 80 | 92 | 86 | 71 | 84 | 87 | 84 |
| **36. Dissolution of Parliament: National Assembly is holding virtual meetings or meets only on bills and Acts related to the state of emergency (14%)** | | | | | | | | | | | | |
| Effectiveness N=4042 (%) | 42 | 43 | 29 | 32 | 44 | 67 | 79 | 40 | 34 | 48 | 58 | 43 |
| Restrictiveness N=943 (%) | 55 | 54 | 74 | 44 | 41 | 35 | 50 | 46 | 52 | 68 | n/a | 54 |
| Compliance n=938 (%) | 52 | 61 | 44 | 64 | 53 | 59 | 85 | 63 | 57 | 68 | 90 | 65 |

| Measure (% affected people)[1] /Variables N (%) | UK | Belgium | Netherlands | Bulgaria | Czech Rep | Finland | India | Latvia | Poland | Romania | Sweden | Mean total |
|---|---|---|---|---|---|---|---|---|---|---|---|---|
| 37. **Penalties or fines for non-compliance with Covid-19 containment measures (13%)** | | | | | | | | | | | | |
| Effectiveness N=5807 (%) | 36 | 52 | 25 | 47 | 46 | 70 | 77 | 54 | 37 | 36 | 52 | 25 |
| Restrictiveness N=920 (%) | 72 | 68 | 89 | 75 | 79 | 60 | 57 | 90 | 86 | 72 | 68 | 89 |
| Compliance N=920 (%) | 64 | 69 | 40 | 79 | 62 | 50 | 87 | 79 | 53 | 64 | 69 | 40 |
| 38. **Mandatory quarantine of citizens returning from abroad (13%)** | | | | | | | | | | | | |
| Effectiveness N=5751 (%) | 58 | 66 | 40 | 69 | 65 | 88 | 88 | 68 | 52 | 73 | 83 | 67 |
| Restrictiveness N=922 (%) | 70 | 58 | 77 | 69 | 70 | 43 | 56 | 82 | 77 | 66 | 35 | 67 |
| Compliance N=920 (%) | 76 | 70 | 43 | 88 | 80 | 75 | 90 | 80 | 73 | 85 | 75 | 82 |
| 39. **Judiciary and courts are closed or hold virtual meetings (12%)** | | | | | | | | | | | | |
| Effectiveness N=4439 (%) | 47 | 47 | 28 | 44 | 50 | 66 | 78 | 47 | 33 | 55 | 63 | 48 |
| Restrictiveness N=849 (%) | 50 | 49 | 70 | 61 | 54 | 75 | 57 | 47 | 75 | 69 | 35 | 62 |
| Compliance N=849 (%) | 77 | 78 | 56 | 90 | 92 | 72 | 88 | 86 | 71 | 80 | 63 | 82 |
| **40. Contact tracing assessment of Covid-19 transmission (9%)** | | | | | | | | | | | | |
| Effectiveness N=1426 (%) | 34 | 55 | 41 | 58 | 46 | 76 | 74 | 65 | 39 | 71 | 72 | 57 |
| Restrictiveness N=176 (%) | 39 | 58 | 62 | 46 | 74 | 47 | 64 | 47 | 57 | 75 | 63 | 60 |
| Compliance N=175 (%) | 51 | 55 | 59 | 84 | 78 | 88 | 79 | 92 | 80 | 82 | 82 | 74 |
| **41. Police forces are allowed to request and obtain citizens' personal information from internet and telephone providers (9%)** | | | | | | | | | | | | |
| Effectiveness N=1983 (%) | 24 | 49 | 7 | 23 | 49 | 54 | 75 | 47 | 15 | 39 | 28 | 37 |
| Restrictiveness N=635 (%) | 88 | 49 | 88 | 88 | 75 | 95 | 64 | 81 | 93 | 85 | 75 | 81 |
| Compliance N=628 (%) | 48 | 66 | 25 | 69 | 59 | 10 | 85 | 68 | 63 | 72 | 43 | 66 |
| **42. Obligatory admission to hospital of seriously ill persons (7%)** | | | | | | | | | | | | |
| Effectiveness N=4092 (%) | 61 | 78 | 52 | 81 | 77 | 87 | 86 | 84 | 62 | 74 | 86 | 77 |
| Restrictiveness N=520 (%) | 56 | 29 | 61 | 34 | 58 | 39 | 56 | 63 | 53 | 60 | 25 | 52 |
| Compliance N=521 (%) | 73 | 71 | 49 | 84 | 94 | 81 | 90 | 76 | 65 | 79 | 71 | 82 |
| **43. Present a health screening form when entering the country (6%)** | | | | | | | | | | | | |
| Effectiveness N=3078 (%) | 48 | 53 | 35 | 48 | 58 | 73 | 84 | 71 | 35 | 66 | 92 | 59 |
| Restrictiveness N=412 (%) | 69 | 56 | 71 | 69 | 69 | 65 | 54 | 76 | 71 | 61 | 0 | 63 |
| Compliance N=412 (%) | 87 | 83 | 64 | 92 | 89 | 55 | 89 | 72 | 71 | 85 | 100 | 86 |
| **44. Mass testing for Covid-19 (i.e. expanded targeted testing or random testing of the general population, irrespective of symptoms) (6%)** | | | | | | | | | | | | |
| Effectiveness N=676 (%) | 31 | 54 | 34 | 42 | 37 | 71 | 74 | 64 | 38 | 64 | 66 | 51 |
| Restrictiveness N=107 (%) | 54 | 76 | 62 | 50 | 76 | 85 | 66 | 22 | 40 | 67 | 37 | 61 |
| Compliance N=108 (%) | 49 | 58 | 66 | 80 | 63 | 90 | 79 | 87 | 40 | 99 | 83 | 71 |
| **Average score for all 44 measures per country** | | | | | | | | | | | | |
| Effectiveness (%) | 48 | 53 | 35 | 55 | 54 | 74 | 77 | 55 | 45 | 59 | 72 | 56 |
| Restrictiveness (%) | 56 | 59 | 65 | 58 | 62 | 40 | 51 | 56 | 59 | 63 | 38 | 57 |
| Compliance (%) | 69 | 74 | 56 | 79 | 74 | 80 | 80 | 75 | 67 | 76 | 78 | 74 |

[1]Measures are ranked from1 to 44 according to their relevance, starting with measures that have affected the highest percent of people personally across countries.

**Table S5. Bivariate Correlations with Compliance (Spearman Rho)**

| Factors ➔<br>Measure | Restrictiveness | Effectiveness | Age | Education | Time News | Trust Hospitals | Trust Government | Reaction Government | Truthful Government | Stress Outbreak | Fear of Infection |
|---|---|---|---|---|---|---|---|---|---|---|---|
| 1. Keep at least 1-2 meters away from other people | -0.3 | 0.6 | 0.1 | 0.1 | 0.1 | 0.1 | 0.3 | -0.3 | 0.1 | 0.04 | 0.3 |
| 2. Cancellation of all mass gatherings and events (cultural, sport, scientific or religious) | -0.2 | 0.5 | 0.03 | 0.1 | 0.1 | 0.2 | 0.3 | -0.3 | 0.2 | 0.06 | 0.3 |
| 3. Recommendation on washing hands more frequently | -0.2 | 0.4 | 0.02 | 0.06 | 0.06 | 0.05 | 0.1 | -0.1 | 0.05 | 0.2 | 0.2 |
| 4. Keeping respiratory hygiene by mandatory wearing of a mask in public places | -0.5 | 0.6 | -0.01 | 0.1 | 0.1 | 0.2 | 0.3 | -0.4 | 0.2 | 0.03 | 0.5 |
| 5. Mandatory stay-at-home, except for essential journeys | -0.2 | 0.5 | 0.1 | 0.1 | 0.1 | 0.1 | 0.3 | -0.3 | 0.2 | 0.1 | 0.3 |
| 6. Recommendation on NOT shaking hands | -0.4 | 0.6 | 0.2 | 0.03 | 0.04 | 0.3 | 0.4 | -0.3 | 0.3 | -0.03 | 0.3 |
| 7. Closure of pubs, cafes, restaurants, except for delivery and takeaway services | -0.1 | 0.4 | -0.06 | 0.1 | 0.1 | 0.1 | 0.2 | -0.2 | 0.1 | 0.1 | 0.2 |
| 8. Closure of cinemas, theaters, opera, concert venues, libraries, museums, heritage sites, discos and/or gambling halls | -0.1 | 0.4 | -0.05 | 0.1 | 0.1 | 0.1 | 0.2 | -0.2 | 0.1 | 0.1 | 0.2 |
| 9. Enhanced cleaning and disinfection procedures | -0.3 | 0.6 | 0.03 | 0.1 | 0.1 | 0.1 | 0.3 | -0.3 | 0.1 | 0.04 | 0.3 |
| 10. Limited number of citizens are allowed to visit open and closed public places, and/or to gather at home | -0.2 | 0.5 | 0.1 | 0.1 | 0.1 | 0.1 | 0.3 | -0.3 | 0.1 | 0.1 | 0.3 |
| 11. Recommendation on coughing or sneezing into your elbow | -0.3 | 0.5 | 0 | 0.02 | 0.01 | 0.1 | 0.2 | -0.1 | 0.1 | 0.1 | 0.1 |
| 12. Recommendation on working from home if possible | -0.1 | 0.4 | -0.1 | 0.1 | 0.1 | -0.01 | 0.1 | -0.2 | 0.02 | 0.1 | 0.2 |
| 13. Declaring state of emergency in the country | -0.1 | 0.4 | -0.003 | 0.1 | 0.1 | 0.1 | 0.2 | -0.2 | 0.2 | 0.1 | 0.3 |
| 14. Closure of non-essential shops and/or large shopping malls | -0.2 | 0.4 | -0.05 | 0.1 | 0.07 | 0.1 | 0.1 | -0.2 | 0.1 | 0.1 | 0.2 |
| 15. A ban on visiting national and nature parks | -0.1 | 0.3 | 0.02 | 0.1 | 0.1 | 0.1 | 0.2 | -0.2 | 0.2 | 0.1 | 0.3 |
| 16. Recommendation on not using public transport (e.g., bus, trains, ships, etc.) | -0.1 | 0.5 | 0.07 | 0.1 | 0.1 | 0.1 | 0.2 | -0.2 | 0.1 | 0.02 | 0.3 |
| 17. Closure of preschools, nurseries and/or primary schools | 0.01 | 0.3 | -0.1 | 0.1 | 0.1 | 0.04 | 0.1 | -0.1 | 0.1 | 0.1 | 0.2 |
| 18. Closure of secondary schools, colleges and/or universities | 0.1 | 0.3 | -0.1 | 0.1 | 0.1 | 0.03 | 0.1 | -0.1 | 0.1 | 0.1 | 0.2 |
| 19. Funerals and weddings are forbidden, or the number of attendees is restricted | -0.1 | 0.5 | -0.02 | 0.1 | 0.1 | 0.1 | 0.2 | -0.2 | 0.1 | 0.1 | 0.3 |
| 20. Closure of indoor and/or outdoor sport facilities | -0.1 | 0.4 | -0.01 | 0.1 | 0.1 | 0.1 | 0.2 | -0.2 | 0.1 | 0.1 | 0.2 |
| 21. Introducing market / shopping hours for vulnerable groups | -0.03 | 0.2 | -0.2 | 0.1 | 0.1 | 0.02 | 0.1 | -0.1 | 0.1 | 0.1 | 0.1 |
| 22. Stopping of all elective medical surgeries and procedures | 0.1 | 0.2 | -0.02 | 0.1 | 0.1 | 0.1 | 0.1 | -0.1 | 0.04 | 0.1 | 0.2 |
| 23. Closure of hotels and tourist accommodation | -0.03 | 0.3 | -0.03 | 0.1 | 0.1 | 0.1 | 0.1 | -0.1 | 0.1 | 0.1 | 0.2 |
| 24. Mandatory reporting of symptoms of illness to health authorities | -0.2 | 0.5 | 0.002 | 0.07 | 0.4 | 0.2 | 0.3 | -0.2 | 0.2 | 0.01 | 0.2 |
| 25. Closure of playgrounds | -0.1 | 0.3 | -0.02 | 0.1 | 0.1 | 0.1 | 0.2 | -0.2 | 0.1 | 0.1 | 0.2 |
| 26. Visits to care homes for the elderly and other at-risk groups are prohibited | -0.1 | 0.4 | -0.1 | 0.1 | 0.1 | 0.1 | 0.2 | -0.14 | -0.03 | 0.1 | 0.2 |
| 27. Final exams are cancelled or postponed | 0.1 | 0.2 | -0.2 | 0.1 | -0.001 | 0.03 | 0.1 | -0.04 | 0.02 | 0.1 | 0.1 |
| 28. Flight suspensions: government bans on flights from or to the country | 0.1 | 0.2 | -0.07 | 0.07 | 0.03 | 0.01 | 0.05 | -0.1 | 0.04 | 0.1 | 0.1 |
| 29. Partial closure of a land border: e.g., people from outside the EU cannot enter the country | -0.001 | 0.2 | -0.1 | 0.2 | 0.1 | 0.03 | 0.1 | -0.1 | 0.02 | 0.08 | 0.1 |
| 30. Using screening procedures such as asking travelers if they have symptoms of flu, have been in contact with someone with flu, performing a visual screen for signs of flu or measuring body temperature | -0.2 | 0.3 | -0.03 | 0.1 | 0.1 | 0.1 | 0.2 | -0.2 | 0.04 | 0.1 | 0.2 |
| 31. Recommendation to quarantine of citizens who belong to high-risk groups or who have been in contact with infected people | -0.1 | 0.5 | -0.01 | 0.1 | 0.1 | 0.1 | 0.2 | -0.2 | 0.04 | 0.03 | 0.2 |
| 32. Introducing a curfew order | -0.1 | 0.3 | 0.07 | 0.1 | 0.1 | 0.1 | 0.2 | -0.1 | 0.2 | 0.1 | 0.2 |
| 33. Total closure of a land border: citizens are not allowed to leave the country | 0.1 | 0.2 | -0.1 | 0.1 | 0.02 | 0.1 | 0.1 | -0.1 | 0.03 | 0.1 | 0.1 |
| 34. Placement of cordon sanitaire on areas with certain infection levels, such as big cities (i.e., a guarded line preventing anyone from leaving the infected area) | -0.05 | 0.1 | -0.01 | 0.06 | 0.1 | 0.1 | 0.2 | -0.1 | 0.2 | 0.1 | 0.1 |
| 35. Mandatory isolation of ill persons for certain periods of time | 0.03 | 0.4 | -0.1 | 0.1 | 0.1 | 0.05 | 0.1 | -0.2 | 0.02 | 0.1 | 0.2 |
| 36. Dissolution of Parliament: National Assembly is holding virtual meetings or meets only on bills and Acts related to the state of emergency | 0.02 | 0.4 | -0.1 | 0.1 | 0.1 | 0.1 | 0.2 | -0.1 | 0.2 | 0.04 | 0.2 |

| Measure (Factors ➔) | Restrictiveness | Effectiveness | Age | Education | Time News | Trust Hospitals | Trust Government | Reaction Government | Truthful Government | Stress Outbreak | Fear of Infection |
|---|---|---|---|---|---|---|---|---|---|---|---|
| 37. Penalties or fines for non-compliance with Covid-19 containment measures | -0.3 | 0.5 | 0.04 | 0.2 | 0.1 | 0.1 | 0.3 | -0.3 | 0.1 | 0.1 | 0.3 |
| 38. Mandatory quarantine of citizens returning from abroad | -0.1 | 0.3 | -0.1 | 0.1 | 0.1 | 0.02 | 0.1 | -0.2 | 0.05 | 0.1 | 0.2 |
| 39. Judiciary and courts are closed or hold virtual meetings | 0.1 | 0.2 | -0.1 | 0.2 | 0.1 | 0.03 | 0.1 | -0.1 | 0.04 | 0.1 | 0.2 |
| 40. Police forces are allowed to request and obtain citizens' personal information from internet and telephone providers | -0.1 | 0.3 | -0.1 | 0.2 | 0.2 | 0.1 | 0.2 | -0.2 | -0.001 | 0.1 | 0.2 |
| 41. Obligatory admission to hospital of seriously ill persons | -0.03 | 0.4 | -0.04 | 0.1 | 0.1 | 0.03 | 0.1 | -0.1 | 0.1 | 0.1 | 0.2 |
| 42. Present a health screening form when entering the country | -0.04 | 0.2 | -0.03 | 0.1 | 0.1 | -0.03 | -0.02 | -0.1 | -0.02 | 0.1 | 0.1 |

**Table S6. Other Factors for Compliance**

**Compliance (mean)**

| Measure (Factors →) | Gender | | Staff | | | Family Infected | | | Health Conditions | | Covid-19 infected | | | | Lost Job | | |
|---|---|---|---|---|---|---|---|---|---|---|---|---|---|---|---|---|---|
| | Female | Male | Medical | Other Essential | Non-Essential | No | Yes | Possibly | No | Yes | No | With COVID-19 symptoms & not been tested | With COVID-19 symptoms & tested positive | Without COVID-19 symptoms & tested positive | No Job Loss | Job Loss with payment | Job Loss without payment |
| **1.** Keep at least 1-2 meters away from other people | 75 | 67 | 77 | 69 | 73 | 72 | 77 | 73 | 71 | 77 | 73 | 68 | 72 | 74 | 74 | 72 | 68 |
| 2. Cancellation of all mass gatherings and events (cultural, sport, scientific or religious) | 83 | 75 | 85 | 77 | 81 | 80 | 86 | 83 | 80 | 83 | 81 | 78 | 86 | 58 | 82 | 80 | 77 |
| 3. Recommendation on washing hands more frequently | 88 | 83 | 88 | 85 | 87 | 87 | 90 | 95 | 86 | 88 | 87 | 84 | 89 | 90 | 87 | 86 | 86 |
| 4. Keeping respiratory hygiene by mandatory wearing of a mask in public places | 75 | 68 | 80 | 68 | 73 | 72 | 81 | 71 | 72 | 76 | 74 | 64 | 75 | 67 | 75 | 68 | 68 |
| 5. Mandatory stay-at-home, except for essential journeys | 78 | 72 | 78 | 74 | 77 | 75 | 83 | 77 | 75 | 80 | 76 | 77 | 85 | 65 | 78 | 76 | 71 |
| 6. Recommendation on NOT shaking hands | 79 | 72 | 84 | 77 | 76 | 76 | 84 | 80 | 75 | 82 | 78 | 74 | 79 | 76 | 80 | 73 | 68 |
| 7. Closure of pubs, cafes, restaurants, except for delivery and takeaway services | 87 | 79 | 86 | 81 | 85 | 84 | 89 | 85 | 85 | 86 | 85 | 82 | 87 | 79 | 86 | 84 | 82 |
| 8. Closure of cinemas, theaters, opera, concert venues, libraries, museums, heritage sites, discos and/or gambling halls | 91 | 84 | 89 | 85 | 89 | 88 | 92 | 90 | 89 | 89 | 89 | 86 | 88 | 79 | 90 | 87 | 86 |
| 9. Enhanced cleaning and disinfection procedures | 77 | 71 | 82 | 74 | 75 | 75 | 79 | 73 | 74 | 80 | 76 | 70 | 70 | 70 | 77 | 75 | 72 |
| 10. Limited number of citizens are allowed to visit open and closed public places, and/or to gather at home | 77 | 69 | 78 | 69 | 75 | 73 | 80 | 77 | 73 | 79 | 75 | 72 | 78 | 68 | 76 | 71 | 70 |
| 11. Recommendation on coughing or sneezing into your elbow | 80 | 76 | 83 | 79 | 78 | 78 | 83 | 80 | 79 | 80 | 79 | 79 | 85 | 69 | 80 | 76 | 77 |
| 12. Recommendation on working from home if possible | 84 | 76 | 68 | 72 | 84 | 81 | 84 | 82 | 82 | 82 | 82 | 79 | 77 | 68 | 83 | 80 | 79 |
| 13. Declaring state of emergency in the country | 86 | 76 | 85 | 79 | 84 | 83 | 88 | 83 | 83 | 85 | 84 | 77 | 88 | 79 | 85 | 84 | 78 |
| 14. Closure of non-essential shops and/or large shopping malls | 87 | 80 | 87 | 79 | 86 | 84 | 89 | 85 | 85 | 85 | 85 | 83 | 82 | 79 | 86 | 84 | 82 |
| 15. A ban on visiting national and nature parks | 76 | 69 | 78 | 70 | 74 | 73 | 80 | 74 | 73 | 76 | 74 | 69 | 80 | 63 | 76 | 72 | 69 |
| 16. Recommendation on not using public transport (e.g., bus, trains, ships, etc.) | 75 | 74 | 82 | 70 | 75 | 74 | 81 | 74 | 73 | 79 | 75 | 72 | 74 | 62 | 76 | 74 | 70 |
| 17. Closure of preschools, nurseries and/or primary schools | 89 | 84 | 87 | 88 | 88 | 87 | 91 | 89 | 88 | 87 | 88 | 87 | 95 | 64 | 88 | 85 | 87 |
| 18. Closure of secondary schools, colleges and/or universities | 90 | 83 | 87 | 88 | 88 | 87 | 91 | 90 | 88 | 87 | 88 | 87 | 94 | 67 | 89 | 84 | 86 |
| 19. Funerals and weddings are forbidden or the number of attendees is restricted | 81 | 73 | 83 | 75 | 77 | 78 | 82 | 82 | 78 | 80 | 79 | 75 | 83 | 41 | 80 | 72 | 76 |
| 20. Closure of indoor and/or outdoor sport facilities | 88 | 79 | 87 | 81 | 85 | 84 | 88 | 88 | 85 | 86 | 85 | 83 | 82 | 73 | 87 | 82 | 81 |
| 21. Introducing market / shopping hours for vulnerable groups | 83 | 76 | 81 | 79 | 81 | 80 | 84 | 83 | 82 | 80 | 81 | 78 | 81 | 56 | 81 | 79 | 82 |
| 22. Stopping of all elective medical surgeries and procedures | 86 | 79 | 84 | 83 | 85 | 84 | 86 | 86 | 84 | 85 | 85 | 84 | 80 | 70 | 85 | 86 | 83 |
| 23. Closure of hotels and tourist accommodation | 88 | 82 | 85 | 83 | 85 | 85 | 89 | 87 | 85 | 86 | 86 | 84 | 82 | 71 | 87 | 85 | 83 |
| 24. Mandatory reporting of symptoms of illness to health authorities | 69 | 67 | 77 | 68 | 67 | 67 | 77 | 66 | 67 | 71 | 69 | 62 | 78 | 70 | 70 | 63 | 65 |
| 25. Closure of playgrounds | 83 | 75 | 80 | 79 | 80 | 80 | 84 | 73 | 80 | 81 | 81 | 73 | 97 | 70 | 81 | 73 | 79 |
| 26. Visits to care homes for the elderly and other at-risk groups are prohibited | 87 | 82 | 87 | 85 | 84 | 85 | 85 | 85 | 84 | 87 | 85 | 83 | 92 | 72 | 86 | 81 | 84 |

| Factors ➔ Measure | Gender | | Staff | | | Family Infected | | | Health Conditions | | Covid-19 infected | | | | Lost Job | | |
|---|---|---|---|---|---|---|---|---|---|---|---|---|---|---|---|---|---|
| | Female | Male | Medical | Other Essential | Non-Essential | No | Yes | Possibly | No | Yes | No | With COVID-19 symptoms & not been tested | With COVID-19 symptoms & tested positive | Without COVID-19 symptoms & tested positive | No Job Loss | Job Loss with payment | Job Loss without payment |
| 27. Final exams are cancelled or postponed | 84 | 78 | 79 | 85 | 82 | 81 | 87 | 85 | 83 | 82 | 82 | 82 | 90 | 50 | 82 | 80 | 82 |
| 28. Flight suspensions: government bans on flights from or to the country | 93 | 84 | 92 | 86 | 90 | 89 | 93 | 87 | 91 | 88 | 90 | 86 | 85 | 85 | 91 | 93 | 88 |
| 29. Partial closure of a land border: e.g. people from outside the EU can’t enter the country | 89 | 80 | 90 | 84 | 86 | 86 | 90 | 84 | 86 | 88 | 87 | 84 | 85 | 80 | 88 | 90 | 83 |
| 30. Using screening procedures such as asking travelers if they have symptoms of flu, have been in contact with someone with flu, performing a visual screen for signs of flu or measuring body temperature | 84 | 80 | 84 | 79 | 81 | 82 | 84 | 83 | 82 | 84 | 83 | 75 | 94 | 83 | 83 | 83 | 79 |
| 31. Recommendation to quarantine of citizens who belong to high risk groups or who have been in contact with infected people | 84 | 79 | 84 | 81 | 82 | 81 | 89 | 76 | 81 | 84 | 83 | 79 | 83 | 93 | 83 | 83 | 80 |
| 32. Introducing a curfew order | 82 | 80 | 83 | 76 | 82 | 80 | 86 | 84 | 80 | 84 | 82 | 70 | 92 | 0 | 82 | 83 | 79 |
| 33. Total closure of a land border: citizens are not allowed to leave the country | 93 | 83 | 90 | 87 | 90 | 90 | 91 | 81 | 90 | 89 | 91 | 81 | 77 | 75 | 90 | 95 | 88 |
| 34. Placement of cordon sanitaire on areas with certain infection levels, such as big cities (i.e. a guarded line preventing anyone from leaving the infected area) | 86 | 80 | 88 | 82 | 83 | 83 | 88 | 84 | 83 | 87 | 84 | 78 | 98 | 53 | 86 | 85 | 79 |
| 35. Mandatory isolation of ill persons for certain periods of time | 85 | 83 | 85 | 83 | 84 | 82 | 90 | 87 | 83 | 86 | 83 | 85 | 95 | 100 | 86 | 86 | 80 |
| 36. Dissolution of Parliament: National Assembly is holding virtual meetings or meets only on bills and Acts related to the state of emergency | 64 | 65 | 65 | 67 | 64 | 64 | 72 | 56 | 64 | 65 | 65 | 61 | 58 | 25 | 66 | 54 | 62 |
| 37. Penalties or fines for non-compliance with Covid-19 containment measures | 72 | 66 | 74 | 64 | 70 | 68 | 77 | 66 | 68 | 73 | 71 | 57 | 66 | 50 | 71 | 65 | 66 |
| 38. Mandatory quarantine of citizens returning from abroad | 85 | 76 | 83 | 78 | 82 | 81 | 89 | 73 | 82 | 80 | 82 | 77 | 74 | 80 | 82 | 82 | 80 |
| 39. Judiciary and courts are closed or hold virtual meetings | 85 | 76 | 83 | 78 | 82 | 81 | 81 | 73 | 82 | 80 | 82 | 77 | 74 | 80 | 82 | 73 | 83 |
| 40. Police forces are allowed to request and obtain citizens’ personal information from internet and telephone providers | 69 | 61 | 63 | 61 | 67 | 66 | 64 | 57 | 66 | 64 | 67 | 52 | 63 | 100 | 66 | 64 | 65 |
| 41. Obligatory admission to hospital of seriously ill persons | 82 | 81 | 89 | 80 | 80 | 81 | 89 | 73 | 81 | 84 | 83 | 66 | 92 | 100 | 83 | 71 | 80 |
| 42. Present a health screening form when entering the country | 87 | 84 | 86 | 81 | 86 | 86 | 85 | 88 | 85 | 87 | 87 | 75 | 94 | 100 | 87 | 89 | 83 |

**Table S7. Multivariable Model factors for Compliance (Stepwise selection, controlling for country effect, only significant associations ($p<0.05$) are reported in the Table)**

**Effect in % Compliance**

| | Measure (Factors →) | Female | Age | Education | Medical staff | Other essential staff | Non-essential staff | Family infected | Covid-19 infected | Effectiveness | Restrictiveness | Time News | Trust Hospitals | Trust Government | Reaction Government | Truthful Government | Stress Outbreak | Fear of Infection | Health Conditions | No Job Loss | Job Loss with payment | Job Loss without payment |
|---|---|---|---|---|---|---|---|---|---|---|---|---|---|---|---|---|---|---|---|---|---|---|
| **1.** | Keep at least 1-2 meters away from other people | 3.5 | 0.1 | | | | | | | 0.5 | -0.1 | | | 0.4 | -0.6 | | 0.5 | 2.1 | | | | |
| **2.** | Cancellation of all mass gatherings and events (cultural, sport, scientific or religious) | 3.4 | | 1.1 | | | | | | 0.4 | 0.02 | | | 0.7 | -0.6 | | 0.7 | 1.9 | | | | |
| **3.** | Recommendation on washing hands more frequently | 2.2 | | | | | | | | 0.3 | -0.1 | | | 0.5 | -0.7 | | 0.5 | 2.2 | 1.2 | | | |
| **4.** | Keeping respiratory hygiene by mandatory wearing of a mask in public places | 4.8 | | | | | | | | 0.4 | -0.1 | | | 1.1 | -1.3 | | 0.2 | 2.3 | | | | |
| 5. | Mandatory stay-at-home, except for essential journeys | 4.9 | 0.1 | 0.7 | | | 2.4 | | | 0.4 | | | | 0.7 | -0.6 | | 0.7 | 2.3 | | | | |
| 6. | Recommendation on NOT shaking hands | 3.5 | 0.2 | | | | -1.7 | | | 0.5 | -0.1 | | | 0.5 | -0.9 | | | 2.8 | | | | -1.9 |
| **7.** | Closure of pubs, cafes, restaurants, except for delivery and takeaway services | 5.2 | -0.1 | 1.3 | -3.1 | | | 2.0 | | 0.3 | 0.03 | | | 0.7 | -0.3 | | 1.0 | | | | | |
| **8.** | Closure of cinemas, theaters, opera, concert venues, libraries, museums, heritage sites, discos and/or gambling halls | 5.0 | -0.1 | 1.4 | | | 2.1 | | | 0.2 | | | | 0.6 | -0.3 | | 0.7 | | | | | |
| **9.** | Enhanced cleaning and disinfection procedures | | | | | | -3.5 | | | 0.5 | -0.05 | | | 0.5 | -0.8 | | | 2.1 | | | | |
| **10.** | Limited number of citizens are allowed to visit open and closed public places, and/or to gather at home | 4.4 | 0.2 | 1.0 | | -2.8 | | 2.7 | | 0.4 | | 0.5 | | 0.7 | -0.7 | | 0.9 | 1.7 | | | | |
| **11.** | Recommendation on coughing or sneezing into your elbow | | | | 2.6 | | | | | 0.4 | -0.1 | | | | -0.3 | | 0.4 | 1.6 | | | | |
| **12.** | Recommendation on working from home if possible | 4.1 | -0.1 | 2.8 | -5.6 | | 9.9 | | | 0.3 | 0.1 | | | | | | | 1.3 | | | | |
| **13.** | Declaring state of emergency in the country | 7.6 | | 1.0 | | | | | | 0.2 | | 0.7 | | 1.0 | -0.9 | | 1.0 | 1.4 | | | | |
| **14.** | Closure of non-essential shops and/or large shopping malls | 5.6 | -0.1 | 1.9 | | | 3.2 | | | 0.2 | | | 0.4 | 0.4 | -0.5 | | 1.1 | | | | | -2.2 |
| **15.** | A ban on visiting national and nature parks | 8.5 | | 1.6 | | | | | | 0.2 | 0.1 | 0.6 | | 1.1 | -1.2 | | 0.8 | 2.7 | | | | -5.3 |
| **16.** | Recommendation on not using public transport (e.g. bus, trains, ships, etc.) | | 0.1 | 0.9 | | | | | | 0.4 | | | | | -0.4 | | 0.5 | 2.1 | | | | |
| **17.** | Closure of preschools, nurseries and/ or primary schools | 3.0 | -0.2 | 1.6 | -3.3 | | | | | 0.3 | 0.1 | | | | | | 0.5 | | | | | |
| **18.** | Closure of secondary schools, colleges and/or universities | 4.5 | -0.3 | 1.0 | -3.3 | | | | | 0.2 | 0.1 | | | 0.5 | | | | 1.6 | | 3.2 | | |
| **19.** | Funerals and weddings are forbidden or the number of attendees is restricted | 6.5 | | 0.7 | | | | | | 0.4 | 0.1 | | | 0.5 | -0.7 | | 0.6 | 1.7 | | | | |
| **20.** | Closure of indoor and/or outdoor sport facilities | 7.3 | | 1.1 | | | 2.6 | 2.8 | | 0.3 | 0.06 | | | 0.7 | -0.7 | | 0.7 | | | | | -4.3 |
| **21.** | Introducing market / shopping hours for vulnerable groups | 6.9 | -0.3 | 0.6 | | | | | | 0.3 | 0.04 | | | 0.8 | -0.7 | | 0.6 | 1.7 | | | | |

| Measure / Factors → | Female | Age | Education | Medical staff | Other essential staff | Non-essential staff | Family infected | Covid-19 infected | Effectiveness | Restrictiveness | Time News | Trust Hospitals | Trust Government | Reaction Government | Truthful Government | Stress Outbreak | Fear of Infection | Health Conditions | No Job Loss | Job Loss with payment | Job Loss without payment |
|---|---|---|---|---|---|---|---|---|---|---|---|---|---|---|---|---|---|---|---|---|---|
| **22.** Stopping of all elective medical surgeries and procedures | 7.9 | | 1.5 | -4.7 | | | | | 0.2 | 0.1 | | | 1.1 | -0.7 | | 0.8 | | | | | |
| **23.** Closure of hotels and tourist accommodation | 5.4 | -0.1 | 1.7 | -5.0 | | | | | 0.2 | 0.1 | | | 0.7 | | | 0.5 | 1.4 | | | | |
| **24.** Mandatory reporting of symptoms of illness to health authorities | | -0.1 | | 6.1 | | | | | 0.4 | | | | 1.1 | -1 | | | 3.7 | | | | |
| **25.** Closure of playgrounds | 7.3 | | 2.4 | | | | | | 0.3 | 0.1 | 0.7 | | 0.6 | -0.9 | | 1.0 | | | | | |
| **26.** Visits to care homes for the elderly and other at-risk groups are prohibited | | -0.1 | 1.2 | | | | | | 0.3 | 0.1 | | | | | | | 1.8 | | | | |
| **27.** Final exams are cancelled or postponed | 4.9 | -0.4 | 2.6 | -6.4 | | | | | 0.2 | 0.2 | | 0.7 | | | | | 1.5 | | | | |
| **28.** Flight suspensions: government bans on flights from or to the country | 6.8 | | 1.2 | | | | | | 0.2 | 0.1 | | | | | | | | | | | |
| **29.** Partial closure of a land border: e.g. people from outside the EU can't enter the country | 7.8 | -0.2 | 2.6 | | | | | | 0.2 | 0.1 | 0.7 | | | | | | | | | | |
| **30.** Using screening procedures such as asking travelers if they have symptoms of flu, have been in contact with someone with flu, performing a visual screen for signs of flu or measuring body temperature | 4.8 | | 1.1 | | | | | | 0.2 | -0.1 | | | | | | 1.0 | | | | | |
| **31.** Recommendation to quarantine of citizens who belong to high risk groups or who have been in contact with infected people | 4.8 | | 1.4 | | | | | | 0.4 | 0.1 | 0.7 | | | -0.5 | | | | | | | |
| **32.** Introducing a curfew order | 6.6 | | | | | | | | 0.2 | | | | 0.9 | -0.8 | | 0.7 | 2.0 | | | | |
| **33.** Total closure of a land border: citizens are not allowed to leave the country | 7.3 | -0.2 | 1.3 | | | 3.8 | | -8.3 | 0.2 | 0.1 | | 0.6 | | | | | | | | | |
| **34.** Placement of cordon sanitaire on areas with certain infection levels, such as big cities (i.e. a guarded line preventing anyone from leaving the infected area) | 6.8 | -0.2 | 1.5 | | | | | | 0.2 | 0.1 | | 1.1 | | -0.6 | | 0.8 | | 4.0 | 4.9 | | |
| **35.** Mandatory isolation of ill persons for certain periods of time | | | 1.5 | | | | 4.7 | | 0.4 | 0.1 | | | | -1.0 | | 0.6 | | | | | -5.4 |
| **36.** Dissolution of Parliament: National Assembly is holding virtual meetings or meets only on bills and Acts related to the state of emergency | | -0.2 | 1.7 | | | | | | 0.4 | 0.1 | 1.1 | | | | | | | | | | |
| **37.** Penalties or fines for non-compliance with Covid-19 containment measures | 0.6 | | 0.3 | | | | | | 0.4 | | | | | -0.1 | | 0.2 | | | | | |
| **38.** Mandatory quarantine of citizens returning from abroad | 9.4 | -0.2 | | | | | | | 0.3 | 0.1 | | | | -0.8 | | 0.7 | | | | | |
| 39. Judiciary and courts are closed or hold virtual meetings | 4.9 | | 3.3 | | | | | | 0.3 | 0.1 | | | | | | 0.8 | | | | | |
| 40. Police forces are allowed to request and obtain citizens' personal information from internet and telephone providers | 10.6 | | 2.4 | | | | | | 0.4 | | | | | | | 2.2 | | | | | |

| Factors ➔<br>Measure | Female | Age | Education | Medical staff | Other essential staff | Non-essential staff | Family infected | Covid-19 infected | Effectiveness | Restrictiveness | Time News | Trust Hospitals | Trust Government | Reaction Government | Truthful Government | Stress Outbreak | Fear of Infection | Health Conditions | No Job Loss | Job Loss with payment | Job Loss without payment |
|---|---|---|---|---|---|---|---|---|---|---|---|---|---|---|---|---|---|---|---|---|---|
| 41. Obligatory admission to hospital of seriously ill persons | | | 1.5 | | | | | | 0.3 | 0.1 | | | | | | | 3.4 | | | | |
| 42. Present a health screening form when entering the country | | | 2.7 | | | | | | 0.2 | | | | | | | 1.3 | | | | | |
